# Inhibition by rno-circRNA-013017 of the apoptosis of motor neurons in anterior horn and descending axonal degeneration in rats after traumatic spinal cord injury

**DOI:** 10.3389/fnins.2022.1065897

**Published:** 2022-12-15

**Authors:** Chuan Qin, Yi Liu, Pei-Pei Xu, Xin Zhang, Zuliyaer Talifu, Jia-Yi Liu, Ying-Li Jing, Fan Bai, Li-Xi Zhao, Yan Yu, Feng Gao, Jian-Jun Li

**Affiliations:** ^1^Department of Urology, Beijing Friendship Hospital, Beijing, China; ^2^School of Rehabilitation Medicine, Capital Medical University, Beijing, China; ^3^China Rehabilitation Science Institute, Beijing, China; ^4^Center of Neural Injury and Repair, Beijing Institute for Brain Disorders, Beijing, China; ^5^Department of Spinal and Neural Functional Reconstruction, China Rehabilitation Research Center, Beijing, China; ^6^Beijing Key Laboratory of Neural Injury and Rehabilitation, Beijing, China; ^7^Department of Rehabilitation Medicine, The Second Hospital of Anhui Medical University, Hefei, China

**Keywords:** circRNAs, apoptosis, axonal degeneration, spinal cord injury, rats

## Abstract

**Introduction:**

Spinal cord injury (SCI) often causes continuous neurological damage to clinical patients. Circular RNAs (circRNAs) are related to a lot of diseases, including SCI. We previously found five candidate circRNAs which were likely to regulate the secondary pathophysiological changes in rat model after traumatic SCI.

**Methods:**

In this study, we first selected and overexpressed target circRNA in rats. We then explored its functional roles using various functional assays in a rat model after SCI.

**Results:**

We found that rno-circRNA-013017—the selected target circRNA—reduced neuron apoptosis, preserved the survival and activity of motor neurons, and regulated apoptosis-related proteins at 3 days post-SCI using western blot, immunofluorescence and polymerase chain reaction. Additionally, we found that rno-circRNA-013017 inhibited descending axonal degeneration and preserved motor neurons and descending axons at 6 weeks post-SCI using immunofluorescence, biotin dextran amine diffusion tensor imaging. Finally, the overexpression of rno-circRNA-013017 promoted the locomotor function of rats after SCI using open-field test and gait analysis.

**Conclusion:**

Focusing on the functions of rno-circRNA-013017, this study provides new options for future studies exploring therapeutic targets and molecular mechanisms for SCI.

## Introduction

The spinal cord is prone to injuries that results in continuous neurological damage to patients, causing a serious social burden and many economic losses (Lin et al., 2019). Spinal cord injury (SCI) is often accompanied by many complications, such as a loss of motor and sensory function, bladder dysfunction, intestinal flora disorders, cardiopulmonary diseases, pressure sores, and sexual dysfunction (Xu et al., 2020). The most important question for SCI patients is whether their motor function can be recovered. In terms of current studies on SCI, some therapies have been reported to produce improvements of motor function, such as myelotomy (Qin et al., 2018), stem cells (Liu X. et al., 2020), and high-dose hormone shock therapy (McCutcheon et al., 2004). However, the therapeutic effects are limited or controversial, and there is no sufficient clinical evidence to prove that these therapies can reverse the motor impairment of SCI. At present, only early surgical decompression and related rehabilitation are widely used as standard clinical interventions for SCI patients.

Spinal cord injury includes primary mechanical injury and subsequent secondary pathophysiological injury. The former is caused by a rapid direct compression and contusion of the spinal cord. The later includes ischemia, apoptosis, oxidative stress, edema, axonal degeneration, and so on (Gedde et al., 2019; Li et al., 2019). These adverse pathological events start from time of injury to several weeks after, and the self-destructive pathological changes extend to the gray and white matter around the injury sites, which can significantly expand the original lesion (Ahmed et al., 2018). The primary injury is in fact mostly unavoidable. Therefore, more attention should be paid to attenuate the secondary pathophysiological injury in the efforts to develop promising treatment for SCI patients (Silvestro et al., 2020). Neuronal apoptosis appears in the early phase after SCI, and is a very important initiating factor in the mechanisms of secondary injury (Ding et al., 2020). The total axons and myelinated axons are reduced immediately after SCI. Especially, motor neurons not only rostral but also caudal to injury site remain viable after injury, which plays an important role in the future functional recovery (Webb and Muir, 2003). The number of myelinated axons in the caudal region of the injury was significantly higher than the lesion site and the rostral region. Moreover, the remaining axons gradually show retrograde degeneration, reaching the peak of degeneration in the chronic phase after SCI (Hassannejad et al., 2019). Notably, the apoptosis of motor neurons and degeneration of descending axons are two important factors related to the impairment of the motor function after SCI, deserving our attention.

Recent advances in studying the functions of non-coding RNAs (ncRNAs), including microRNAs (miRNAs), long non-coding RNAs (lincRNAs), and circular RNAs (circRNAs), may be at the point of breaking this impasse. Due to different functions, ncRNAs are upregulated or downregulated after SCI. More and more studies in this field have identified some ncRNAs with abnormal expressions indicating neurological damage can be alleviated by normalizing the expression levels of certain lncRNAs and miRNAs in rats after SCI (Hwang et al., 2016). For instance (Zhu et al., 2017), one microarray data found 14 miRNAs were upregulated and 46 miRNAs were downregulated by 2 times compared with sham rat spinal cords, and miR-494 was one of the miRNAs being most significantly downregulated and improves functional recovery and inhibits apoptosis by modulating PTEN/AKT/mTOR pathway in rats after spinal cord injury. CircRNAs are a special type of endogenous non-coding RNAs formed by back-splicing events *via* protein-coding exons, and have attracted a wealth of interest because mounting evidence has found the altered expression of specific circRNAs play an important role in human diseases (Qu et al., 2015; Bai et al., 2018). In our previous study (Qin et al., 2019), we found an altered circRNA expression pattern which may be involved in physiological and pathological processes in rats after traumatic SCI, providing deep insights into numerous possibilities for SCI treatment targeting by regulating circRNAs. These results are in line with several other studies focusing on circRNAs and SCI (Wu et al., 2019; Liu Y. et al., 2020; Peng et al., 2020). However, studies focusing on the physiological functions of circRNAs in SCI are very limited. Only one recent study found that circ-HIPK3 relieved neuronal cell apoptosis by regulating the miR-588/DPYSL5 axis in a mostly *in vitro* SCI model (Zhao J. et al., 2020). As a result, based on our previous work, the aim of this investigation was to determine the potential functional roles of candidate circRNAs in the apoptosis of motor neurons and degeneration of descending axons after traumatic SCI. These findings may provide new clues for study of the functions of circRNA underlying SCI, and novel molecular targets for the clinical therapy of SCI.

In the present study, we first selected target circRNA *via* three methods from the five candidate genes identified in our previous study (Qin et al., 2019). Subsequently, we performed an intrathecal injection of target circRNA overexpressed by adeno-associated virus (AAV) into the spinal cord of rats before injury to achieve pre-protection. We then conducted molecular biology experiments to identify the functional roles of target circRNA in the apoptosis of motor neurons in the anterior horn and descending axonal degeneration in rats after SCI. Lastly, we used an open-field test and gait analysis to evaluate the behavior of rats at different time points after injury.

## Materials and methods

### Animals and experimental groups

This study was performed in accordance with the principles of the Basel Declaration and the recommendations of the National Institute of Health Guide for the Care and Use of Laboratory Animals (NIH Publications No. 8023, revised 1978). The protocol was approved by the Institutional Animal Care and Use Committee of Capital Medical University, Beijing, China. Female Sprague–Dawley rats (age: 10–12 weeks; weight: 250–300 g) were purchased from the Animal Care Center of the Academy of Military Medical Sciences (Beijing, China). Rats were housed in a temperature-controlled (20–28°C) and light-controlled (12 h light/dark cycle) room. They were habituated to the housing conditions for at least 7 days before SCI. Additionally, the animals had free access to standard rat chow and tap water; however, food was withheld overnight before surgery. All these procedures were referred to our previous study (Qin et al., 2019).

Rats were randomly assigned to four groups using a computer-generated randomization schedule: The rats in the sham control group underwent laminectomy alone, without contusion; the rats in the SCI group underwent laminectomy and were subjected to contusion; the rats in the AAV-013017 + SCI group underwent an intrathecal injection of the target gene in AAV before injury and were then subjected to contusion; and the rats in the AAV-NC + SCI group underwent an intrathecal injection of a negative control in AAV and were then subjected to contusion. The operators performing the surgeries were blinded to the experimental groups.

### Selection of target circular RNA

Five candidate circRNAs closely related to pathophysiology after SCI were selected in our previous study (Qin et al., 2019). In this investigation, one target circRNA was chosen from candidates *via* three methods: (1) Bioinformatics were used to predict the interactions between a certain circRNA and its target genes (miRNA/mRNA); (2) a literature review was used to obtain evidence that target genes of the circRNA were involved in apoptosis after SCI in a rat model; and (3) the luciferase assay was performed, where luciferase constructs containing either base pairs of the rno-miR-16-5p 3′ UTR or two base-pair regions containing the predicted binding sites and a second construct, containing site-directed mutations in the predicted binding site, were prepared using methods as previously described (Qin et al., 2020). The rno-miR-16-5p 3′ UTR, including the predicted binding sites for rno-circRNA-013017, was cloned into a psiCheck-2 dual-luciferase vector. Mutant rno-miR-16-5p 3′ UTR luciferase vectors were produced in the predicted rno-circRNA-013017 binding regions. These plasmids and a rno-circRNA-013017 mimic were co-transfected into HEK293T cells. Finally, luciferase assays were conducted using the dual-luciferase system (Promega, Madison, WI, USA), according to the manufacturer’s instructions.

### Adeno associated virus production and intrathecal injection

The AAV-PHP.EB subtype and pK25ssAAV plasmid were used in our study (GENESEED, Guangzhou, China) and the final concentration of AAV was 1 × 10 e13 viral gene copies/μL. First, vector construction was performed (rno_circRNA_013017-F: cgGAATTCTAATACTTTCAGG TC CACAACTCCCAGGCCTGTT. rno_circRNA_013017-R: cgG GATCCAGTTGTTC TTACCTTG TCTCAGGGTTGTAGCC TA). Then, cell transfection was performed. 293T cells were transfected into C13017, NC (negative control, PK25SSAAV-CIR) and control group, and the cells were collected after 40 h. AAV-NC means AAV with transfected empty plasmid without target gene, compared to AAV with target gene. Next, in the 293T cell line, rno-circRNA-013017 was overexpressed by over 1,000 times after gene transfection using PCR (shown in Supplementary Table 1). GAPDH was used as housekeeping gene. The lateral sites of the spinal cord were intrathecally injected with either a circControl-GFP AAV (transfected empty plasmid) or circ-013017-GFP AAV using the following procedure: In brief, 3 weeks before SCI, all rats were anesthetized *via* an intraperitoneal injection of sodium pentobarbital solution at 40 mg/kg body weight, and their backs were then shaved and sterilized. After holding or fixing the rats in a stereotaxic frame, a 2 cm longitudinal midline incision was made to expose the spinous processes of the thoracic (T)10 vertebrae. Following stripping of the paraspinal muscles, partial laminectomy was performed at the T10 level to expose the lateral spinal dura mater, without tearing it, in sterile conditions. Rats were fixed in the prone position on a stereotaxic instrument (stoelting, USA). At the same time, we placed the microinjection needle (Hamilton 7000, Switzerland) perpendicular to the injection pump above the T10 spinal cord. We then slowly penetrated the meninges by about 1mm and then injected. The rats were not violently shaken. Lateral sites of the cord were selected as injection points and the central vessel was avoided. A total of 2 μL of AAV-circRNA-013017 or AAV-NC for each site was injected at a rate of 1 μL/min, without obvious abnormality resulting in the rats. After each injection, the needle was kept in place for 3 min before pulling it out, in order to prevent the leakage of AAV and cerebrospinal fluid. The wound was then sutured in layers. After anesthesia, if there was abnormal behavior of both lower limbs of rats, the test was ineligible and unaccepted. Three weeks later, rats with pre-protection of AAV were subjected to SCI contusion.

### Contusion spinal cord injury model and tissue collection

In this study, the contusion injury method was adopted to trigger moderate injuries in rat models. For rats in the AAV-013017 + SCI and AAV-NC + SCI groups, the wound was reopened 3 weeks after the intrathecal injection. In order to maintain the consistency and continuity of research, all procedures of surgical operation were based on standard protocols and consistent with our previous study, briefly, all rats were anesthetized by intraperitoneal injection of 0.4 mg/g body weight 10% chloral hydrate (Kermel, Tianjing, China), and their backs were shaved and sterilized. After suspending the rats in a stereotaxic frame, a 4-cm-long longitudinal midline incision was made to expose the T9–T11 spinal column. Following stripping of the paraspinal muscles, laminectomy was performed at the T10 level to expose the spinal dura mater without tearing it in a sterile condition. Subsequently, rats were clamped by their spinous processes at T9 and T11 with sterilizing forceps, which was followed by spinal cord contusions induced by an Infinite Horizon Impactor (IH-0400 Impactor, Precision Systems and Instrumentation, LLC, United States), leading to a moderate injury at the T10 level. For parameter setting, a standard rat tip impactor size (2.5 mm in diameter), programmable dwell time (1 s) and programmable force levels (225 kDynes) were applied to induce moderate intensity injuries. Errors greater than 3% in terms of force levels and fracture of spinal dura mater were not accepted. Finally, the wound was then sutured in layers. A warm environment was established to maintain body temperature during surgery. The cord surface showed signs of subarachnoid hematoma and an intense dark brown/purple color. The rats with swinging tails that quickly retracted their lower limbs immediately after SCI were regarded as eligible as described previously (Qin et al., 2019). Notably, 4 mL of Ringer lactate solution was administered intraperitoneally to supplement electrolytes and body fluids. Rats were then housed in individual cages. Animals were fed with free access to food and water. Penicillin (40,000 U, intramuscular injection) was administered daily for 3 days to prevent systemic infection. The bladders were emptied manually every 8 h until the rats were killed.

For PCR and the WB assay, rats in all groups were euthanized with an overdose of 40 mg/kg sodium pentobarbital solution, and a 1 cm long segment of spinal cord, including the injury epicenter (T10) as well as adjacent rostral and caudal segment of spinal cord (T9 and T11) was quickly dissected and collected without perfusion in advance and was then fresh-frozen in liquid nitrogen to prevent RNA degradation.

For TUNEL, IF, Nissl’s staining, and BDA, rats in all groups were euthanized with an overdose of 40 mg/kg sodium pentobarbital solution, Subsequently, they were transcardially perfused with 250 mL of 0.9% NaCl (4°C), followed by 500 mL of 4% paraformaldehyde (PFA) (4°C) in 0.1 M phosphate-buffered saline (PBS, pH 7.4). A 1.5 cm segment of spinal cord, including the injury epicenter and caudal and rostral segment, was then dissected for pathological experiments.

### Quantitative reverse-transcription polymerase chain reaction assay

Three days after SCI, the PCR assay was performed to detect the expression level of rno-circRNA-013017 in rats of all groups (*n* = 5 per group). As previously reported (Qin et al., 2020), the total RNA from 10 mm long spinal cord segments containing the injury epicenter was extracted with TRIzol, according to the manufacturer’s standard protocols (Invitrogen, Carlsbad, CA, USA), we synthesized cDNA. Quantitative reverse-transcription polymerase chain reaction (qRT-PCR) was performed in a ViiA 7 Real-time PCR System (Applied Biosystems) with PCR master mix (2×, Arraystar). The parameter settings were 95°C denaturation (10 min), 95°C (10 s), and 60°C (60 s), which was repeated for 40 cycles. After the amplification reaction was finished, the procedure was performed as follows: 95°C (10 s), 60°C (60 s), and 95°C (15 s). Glyceraldehyde 3-phosphate dehydrogenase (GAPDH) acted as an internal control to normalize the data. All reactions were performed in triplicate. The relative expression levels of circRNAs were calculated using the relative standard curve method (Larionov et al., 2005). The primers in PCR were used as previously described (Qin et al., 2019). The primers and corresponding information designed for target circRNA (rno_circRNA_013017: F-5′ ATATTTGCTGCTCGTGAATTTA3′; R-5′TGGGAGTTGTGG ACCTTGT 3′; Ta OPT 60°C; Product length 88bp).

### Western blot analysis

After the rats (*n* = 4 per group) were sacrificed, segments of spinal cord (1 cm) were isolated using the lesion site as the epicenter, and spinal cord tissues were homogenized in a lysis buffer containing 1% Non-idet P-40, 20 mM Tris, pH 8.0, 137 mM NaCl, 0.5 mM EDTA, 10% glycerol, 10 mM Na2P2O7, 10 mM NaF, 1 mg/mL aprotinin, 10 mg/mL leupeptin, 1 mM vanadate, and 1 mM phenylmethylsulfonyl fluoride. Tissue homogenates were incubated for 20 min at 4°C and centrifuged at 25,000 g for 30 min at 4°C. Protein concentrations were determined using a bicinchoninic acid (BCA) protein assay kit (Beyotime, Shanghai, China). Samples (30 mg of protein) were electrophoresed onto a 12% sodium dodecyl sulfate/polyacrylamide gel (SDS/PAGE), and transferred to PVDF membranes (Millipore, Mississauga, Canada). The membranes were blocked in 5% non-fat milk for 1 h at RT. The membranes were then incubated with polyclonal antibodies against Bax (1:1000), Bcl-2 (1:1000), and Bcl-w (1:1000), which were purchased from Cell Signaling Technology, Inc. (USA). β-actin (1:1000, Cell Signaling Technology, USA) was used as an internal control for protein loading. The membranes were processed with horseradish-peroxidase-conjugated secondary antibody (1:2000; Abcam). The film signals were digitally scanned and then quantified using the Image J software (National Institutes of Health, USA). Experiments were repeated three times, and the values obtained for the relative intensity were subjected to statistical analysis. Background in films was subtracted from the optical density measurements.

### Nissl’s staining

At 3 days or 6 weeks after SCI, a 1.5 cm segment of spinal cord, including the injury epicenter and caudal and rostral segments, was dissected and postfixed in the same fixative for 24 h at 4°C (*n* = 5 per group). After fixation, the tissue blocks were embedded in paraffin. Transverse sections (10 μm thickness) were taken through the middle of the spinal lesion site and adjacent segments, which were defined as the epicenter, rostral, and caudal, and placed on Superfrost Plus Slides (Thermo Fisher Scientific, Bremen, Germany). The samples were stained with Nissl staining solution (methylene blue, Solarbio, Beijing, China), immediately followed by the administration of one to two drops of permanent mounting medium, and covered with a glass coverslip. All sections were digitally scanned at a high resolution using HistoFAXS 3.0 (Tissue Gnostics, Vienna, Austria). The acquired images were viewed using the associated proprietary viewing software (FAXS viewer, Tissue Gnostics, Vienna, Austria). Photoshop CC (Adobe, San Jose, CA, USA) and ImageJ (National Institutes of Health, Bethesda, MD, USA) software was used to process and analyze images.

### Terminal deoxynucleotidyl transferase dUTP nick-end labeling

At 3 days after SCI, to detect apoptosis, transverse sections (10 μm thickness) were taken through the middle of the spinal lesion site and adjacent segment, which were defined as the epicenter, and the rostral and caudal that were obtained in the above experimental procedure were further subjected to TUNEL staining (*n* = 5 per group). TUNEL was performed according to the instructions of the manufacturer (Roche, South San Francisco, USA), as described previously (Zhu et al., 2017). Briefly, slides were dewaxed in xylene, rehydrated in graded alcohols, and placed in dH2O. These slides were then incubated for 15 min at RT with 20 μg/mL Proteinase K (Gibco BRL, Gaithersburg, MD, USA). The slides were rinsed twice with PBS before being incubated in TUNEL reaction mixture for 60 min at 37°C. After being rinsed with PBS three times for 2 min, sections were incubated with HRP-streptavidin reagent (1:200) in PBS for 30 min at RT. After being rinsed with PBS three times for 5 min, sections were counterstained with DAPI. Then, sections were treated with anti-fluorescence quenching mounting solution. All sections were digitally scanned at a high resolution using HistoFAXS 3.0 (Tissue Gnostics, Vienna, Austria). The acquired images were viewed using the 3.3.6 version of Case Viewer (The Digital Pathology Company, Hungary).

### Immunofluorescence

At 3 days or 6 weeks after SCI, a 1.5 cm segment of the spinal cord, including the injury epicenter and caudal and rostral segments, was dissected and paraffin-embedded (*n* = 5 per group). Transverse sections (10 μm thickness) were taken through the middle of the spinal lesion site and adjacent segment, which were defined as the epicenter, rostral, and caudal. All sections were dewaxed, vacuum negative pressure treatment with 0.3%H_2_O_2_ methanol for 5 min, washed by distilled water, vacuum negative pressure treatment with 0.01M citrate buffer, PBS washing for 3 times for antigen retrieval and permeabilized with 0.3% Triton X-100 in PBS for 15 min. After being blocked with 10% normal goat serum (NGS) in 0.3% Triton X-100 for 1 h, sections were incubated overnight with primary antibodies, including anti-NeuN (ab177487, 1:500, Abcam, Cambridge, MA, USA), a neuronal marker; anti-choline acetyltransferase (ChAt, ab178850, 1:2000, Abcam, Cambridge, MA, USA), a marker for motor neurons; and anti-GFP (ab13970, 1:500, Abcam, Cambridge, MA, USA), a labeled protein for transfection. The following day, slides were washed 3 × PBS 0.1% Triton X-100 for 5 min, incubated with Alexa-Fluor-conjugated antibodies (Alexa Fluor^®^ 488 for NeuN and GFP and 647 for ChAt, 1:500, Abcam, Cambridge, MA, USA), counterstained with DAPI, and mounted on poly-L-lysine-coated glass slides. All sections were examined by fluorescence microscopy.

### Biotin dextran amine

At 5 weeks after SCI (1 week before tissue collection), a microinjection of biotin dextran amine (BDA) (10%, D22913, MW 10,000 kDa; Invitrogen, USA) was given to rats in all groups to label descending propriospinal axons and possibly other axons of the passage (*n* = 5 per group). The surgical procedure was similar to the intrathecal injection of AAV. In brief, the injection point was a depth of 1 mm, 1 mm lateral to the midline of T8 after partial laminotomy (1 μL for each site, 2 μL in total). After awakening, if there was abnormal behavior of both lower limbs in rats, it was considered that the secondary injury was too heavy for rats, and they did not qualify for inclusion in the test. One week later, 1.5 cm of the spinal cord, including T9–T11 segments (defined as rostral, epicenter, and caudal)in all rats were collected after cardiac perfusion. In brief, after 20 and 30% sucrose solution gradient dehydration and OCT embedding, spinal cord tissue was frozen and sliced in the sagittal plane with a thickness of 30 μm. Additionally, tissue sections were fixed after 4% PFA, and were incubated in a mixed solution of 0.3% H2O2 and 50% ethanol for 10 min, 0.1 mol/L glycine for 10 min, and a block solution containing 0.5% blocking reagent for 30 min. All sections were incubated in strepavidin-HRP (1:500) at 4°C overnight and the amplified solution containing Tyramide (1:250) at room temperature for 5 min, and were mounted on poly-L-lysine-coated glass slides. All sections were examined by fluorescence microscopy.

### Diffusion tensor imaging

At 6 weeks after SCI, we used 7.0 T MRI to perform diffusion tensor imaging (DTI) scans of the spinal cord tissue of rats in each group (*n* = 5 per group). The DTI scan was performed in the Imaging Laboratory of Capital Medical University. The animals were under anesthesia during the entire scanning process, prone in the coil of the nuclear magnetic equipment. The scanning sequence included T2-weighted imaging (T2WI) and DTI scanning. DTI scanning used the following parameters: single-spin-echo planar imaging (SE-EPI); TR/TE = 6250/22 ms; FOV = 4 × 4 cm; matrix = 128 × 128; scan time: 15 min; layer thickness: 1 mm; diffusion weighting coefficient *b*-value: 1000 s/mm^2^; and diffusion-sensitive gradient in 30 directions, covering the damaged area, and defining the white-matter centerline on the T2-weighted image. We segmented the T2 image into the entire spinal cord, gray matter, and white matter, and then used it as a mask for DTI processing. DTI image preprocessing included the following: (1) The registration of T2W images; (2) correction of the eddy current; and (3) calculation of the following DTI parameters: FA (FA is a summary measure of microstructural integrity. While FA is highly sensitive to microstructural changes, it is less specific to the type of change) and AD (AD tends to be variable in WM changes and pathology. In axonal injury AD decreases). The epicenter (T10), rostral cord (T9), and caudal cord (T11) were defined as the regions of interest (ROI). During the ROI selection process, the cavity volume (zero signal intensity) and partial volume effects of cerebrospinal fluid were avoided. The data were processed using the system’s analysis software and SPSS analysis was performed.

### Evaluation of the motor function

The locomotor function was evaluated based on the open-field test (Basso Beattie Bresnahan, BBB) and gait analysis. For the open field test, the BBB score was used for all rats (*n* = 5 per group) at 1 days, 3 days, 1 week, 2 weeks, 3 weeks, 4 weeks, and 6 weeks postinjury. We made a square hard board with a length of 50 cm and a baffle with a height of 20 cm for the test. Each rat was tested for 4 min, without human intervention. The performances of the left and right hindlimbs of rats were rated separately and averaged to generate the BBB scores. A normal function was rated as 21 points, and a lower score reflected a more impaired locomotor function (Scheff et al., 2002). Our two experienced researchers who performed this test were blinded to the experimental groups. The final score was given by a consensus. Next, at 6 weeks post injury, gait analyses for rats of all groups were performed. Specific parameters of locomotion were quantified using the DigiGait Image Analysis System (Cabezos et al., 2008). Rats were pretrained at a speed of 21 cm/s for at least 15 min each day before SCI for 7 days, and then tested at a speed of 18 cm/s. For each test, at least five complete step cycles were recorded. A high-speed digital camera captured the movement of each paw and the footage was then analyzed using the Digigait analysis software (Digigait 12.4). The entire experiment was conducted in a dark room. Operators performing gait analysis were blind to the experiments. The analysis parameters included the swing/stride, stance/stride, swing/stance, stride length, stride frequency, and paw area.

### Statistical analysis

**FIGURE 1:**
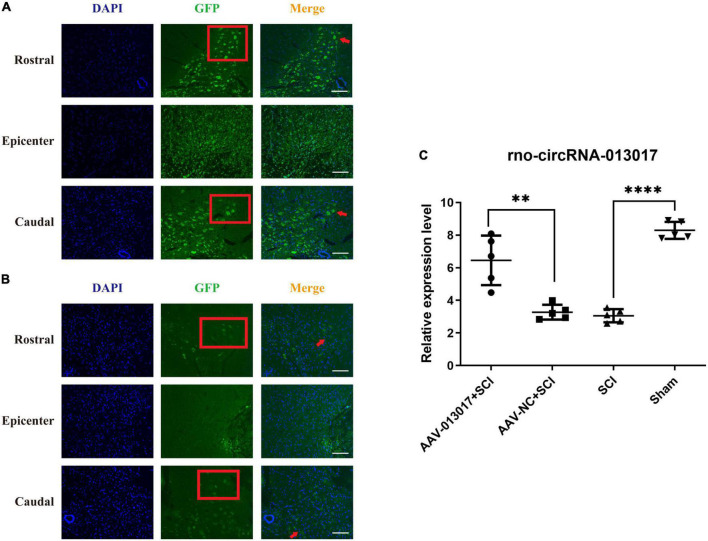
Overexpression of rno_circRNA_013017 in the spinal cords of rats after an adeno-associated virus (AAV) microinjection at 3 days after spinal cord injury (SCI). **(A)** Expression of GFP in the AAV-013017 + SCI group was detected by immunofluorescence (IF) staining. Motor neurons in the anterior horn area are indicated by a red arrow (*n* = 5 per group). **(B)** Expression of GFP in the AAV-NC + SCI group, as detected by IF staining. Motor neurons in the anterior horn area are indicated by a red arrow (*n* = 5 per group). **(C)** The rno_circRNA_013017 expression was significantly altered in rats of all groups (*n* = 5 per group). AAV-NC (negative control) means AAV with transfected empty plasmid without target gene, compared to AAV with target gene. ****Indicates *p* < 0.0001; **indicates *p* < 0.01.

Statistical analysis was performed using SPSS software (version 21.0, Chicago, IL, United States). The data were shown as the mean ± standard deviation (SD). The histogram was produced by GraphPad Prism 8. All data were subject to tests for normality using Shapiro-Wilk test. Most data exhibit a normal/Gaussian distribution. And the few data that do not exhibit a normal/Gaussian distribution were analyzed *via* a non-parametric equivalent. For example, in Figure 1C, the PCR data were analyzed by Kruskal-Wallis. Student’s *t*-tests were used to determine significance between two groups, such as SCI vs sham and AAV-013017 + SCI vs AAV-NC + SCI. One-way analysis of variance followed by *post-hoc* Tukey’s analysis was performed to compare groups of three or more, such as SCI vs AAV-013017 + SCI vs AAV-NC + SCI. For BBB score analysis, the repeated measures ANOVA was used. *P* < 0.05 was used to be statistically significant.

## Results

### Selection of target circular RNA and study design illustration

In our previous study (Qin et al., 2019), after microarray scanning and normalization, the dysregulated expression of all 13279 circRNAs between groups was detected. A total of 1676 circRNAs were differentially expressed (fold change ≥ 2; *P* < 0.05) between the groups, 1261 of which were significantly downregulated and 415 of which were significantly upregulated in the SCI group (Supplementary Figures 1A,B). Next, five circRNAs with relatively high fold changes and similar tissue sample distributions within each group were randomly selected for low-throughput verification with qRT-PCR. Five candidate circRNAs, namely, rno_circRNA_005342, rno_circRNA_015513, rno_circRNA_00 2948, rno_circRNA_006096, and rno_circRNA_013017 were found to be significantly downregulated and involved in physiological and pathological processes in rats after SCI. Next, we needed to perform functional verification of one target circRNA selected from these candidates. First, the circRNA/miRNA/mRNA interactions were predicted in combination with in-house miRNA target prediction software based on TargetScan and miRanda software. An entire network of these interactions was depicted. The results showed that a total of 5 circRNAs, 60 miRNAs and 253 mRNAs were included, presenting a large interaction network. CircRNAs can serve as ceRNA for miRNAs, and miRNAs usually inhibit target mRNAs (Supplementary Figure 1C), using bioinformatics, we found that rno_circRNA_013017 and its target genes rno-miR-16-5p/bcl-2 had a high prediction score. The rno_circRNA_013017/rno-miR-16-5p/bcl-2 network displayed a high potential interaction by prediction (Supplementary Figure 2A). A literature search was conducted, and we learned that some researchers previously found that rno-miR-16-5p/bcl-2, which are target genes, regulate the apoptosis process in different diseases. In a rat model subjected to SCI, one study showed that the inhibition of rno-miR-16-5p negatively regulated bcl-2, leading to its upregulation, thus inhibiting apoptosis after SCI (Zhao Q. C. et al., 2020). This evidence suggests that the downregulation of rno_circRNA_013017 might upregulate rno-miR-16-5p, thus promoting apoptosis after SCI. Third, using luciferase reporter assay, a sequence alignment of the rno_circRNA_013017 with the 3′UTR of the rno-miR-16-5p was shown and the expression of rno-miR-16-5p was inhibited by the rno_circRNA_013017 directly targeting its 3′UTR (Supplementary Figures 2B,C). This lays the foundation for future research.

The study design is presented in Supplementary Figures 2D,E. In brief, in terms of apoptosis detection, we first administrated AAV-circRNA-013017 or AAV-control to rats *via* an intrathecal injection at 3 weeks before SCI modeling—that is, pre-protection. Three weeks later, an SCI contusion model was successfully established for all rats except those in the sham group. At 3 days after injury, we collected tissues and performed apoptosis-related experiments. Three weeks later, an SCI contusion model was established. At 5 weeks after injury, biotin dextran amine (BDA) was administrated to each rat in all groups to mark the descending axons. One week later, rats were subjected to diffusion tensor imaging (DTI) scan and gait analysis and then sacrificed for relative experiments at 6 weeks post injury. At different time points after SCI, the BBB score was calculated to evaluate the locomotor function of rats in all groups.

### Overexpression of rno_circRNA_013017 in the spinal cords of rats after an adeno associated virus injection

In this part of the study, we used immunofluorescence (IF) and a polymerase chain reaction (PCR) to determine whether rno_circRNA_013017 was overexpressed after microinjecting AAV-Green Fluorescent Protein (GFP) at 3 days after SCI. First, in terms of IF (Figures 1A,B), in the AAV-013017 + SCI and AAV-NC + SCI groups, we found that the GFP marker was positive in motor neurons in the anterior horn area, as indicated by the red arrow, especially in the rostral and caudal adjacent segments of the epicenter. That means the ventral horn faces the top of figure. The Red frame showed the motor neurons in ventral horn and the results showed more neurons marked by GFP in rostral area than caudal area. However, in the injury epicenter (T10), few motor neurons marked by GFP in the anterior horn area were seen. Instead, some nerve cells were found in the epicenter, which was likely caused by the abnormal microenvironment of edema, necrosis, and rupture in the acute phase after injury. Second, in terms of PCR (Figure 1C), at 3 days after injury, we found that rno_circRNA_013017 was differentially expressed in different groups. Additionally, the expression of the target gene in the AAV-013017 + SCI group was significantly higher than in controls (*p* = 0.002). Moreover, in this study, we found that the target gene’s expression was significantly upregulated in the sham group in comparison to the SCI group (*p* < 0.0001), which was in line with the results presented in our previous work (Qin et al., 2019). Altogether, these results demonstrated that rno-circRNA-013017 was overexpressed after an AAV injection, which laid the foundation for the verification of subsequent functional experiments.

### Inhibition by rno-circRNA-013017 of the apoptosis of motor neurons in the spinal anterior horn

We used terminal deoxynucleotidyl transferase dUTP nick-end labeling (TUNEL), IF, Nissl’s staining, and Western blotting (WB) to identify the inhibiting effect of overexpressed rno-circRNA-013017 on the apoptosis of motor neurons in the spinal anterior horn at 3 days after SCI.

**FIGURE 2:**
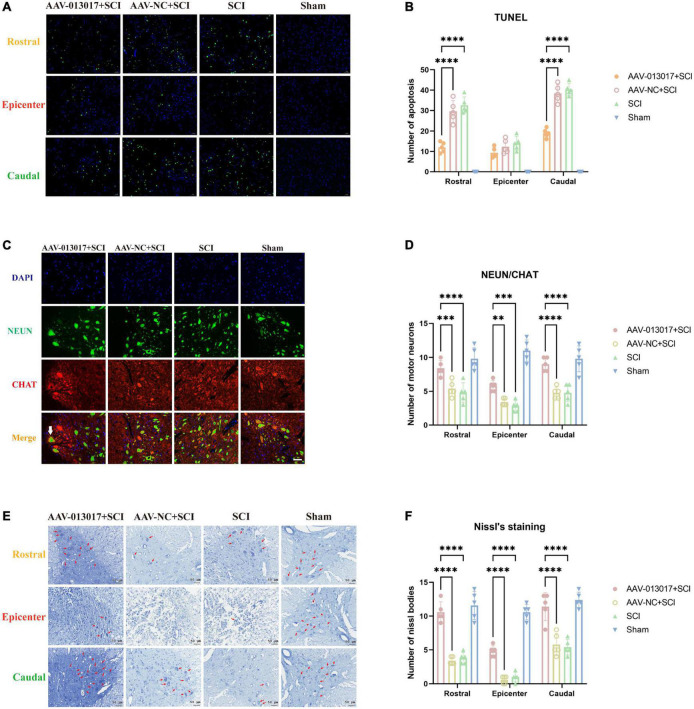
Inhibition by rno-circRNA-013017 of the apoptosis of motor neurons in the spinal anterior horn at 3 days after SCI. **(A)** In the rostral and caudal adjacent segments and epicenter, apoptotic neurons in the anterior horn were identified by terminal deoxynucleotidyl transferase dUTP nick-end labeling (TUNEL), as indicated by the green points (*n* = 5 per group). **(B)** In the rostral adjacent segment, neurons marked by NeuN and motor neurons marked by ChAt in the anterior horn area were shown in all groups. The white arrow indicates a typical motor neuron with an irregular cell body, nucleus, axon, dendrite, and other cell structures (*n* = 5 per group). **(C)** In the epicenter, few normal neurons and motor neurons in the anterior horn area were seen in the three groups subjected to SCI (*n* = 5 per group). **(D)** In the caudal adjacent segment, neurons marked by NeuN and motor neurons marked by ChAt in the anterior horn area were shown in all groups. The white arrow indicates a typical motor neuron with an irregular cell body, nucleus, axon, dendrite, and other cell structures (*n* = 5 per group). **(E)** Nissl bodies, indicated by red arrows, in the anterior horn area were seen in all groups (*n* = 5 per group). AAV-NC (negtive control) means AAV with transfected empty plasmid without target gene, compared to AAV with target gene. **(F)** Number of nissl bodies. ****Indicates *p* < 0.0001; ***indicates *p* < 0.001; **indicates *p* < 0.01.

First, after TUNEL detection (Figure 2A) for comparisons among each segment of different groups, the neurons in the sham group were basically free of apoptosis, which was in accordance with surgical operations. In the rostral and caudal adjacent segment of the epicenter, apoptotic neurons in the anterior horn were the least numerous in the AAV-013017 + SCI group after SCI, as indicated by the green points. However, in the epicenter, there were no significant differences of neuron apoptosis among the three groups subjected to SCI, which was likely related to the degree of injury, time of tissue collection, and peak time of neuronal apoptosis. Notably, for different segments of each group, neuronal apoptosis was more significant in T9 than T11 after SCI. As seen in quantitative figure (Figure 2B), apoptotic cells in AAV-013017 + SCI group were least after SCI in TUNEL detection in rostral and caudal segment significantly (*p* < 0.0001). However, in epicenter cord, no significant differences were found between groups.

Second, as shown in the figures of IF (Figures 2C,D and Supplementary Figures 3A,B), in the rostral and caudal adjacent segments, more neurons marked by NeuN and motor neurons marked by ChAt in the anterior horn area were seen in the AAV-013017 + SCI group than in the AAV-NC + SCI and SCI groups. The white arrow indicates a typical motor neuron with an irregular cell body, nucleus, axon, dendrite, and other cell structures. Notably, as NeuN selectively stained alpha-motor neurons but not-gamma motor neurons (Friese et al., 2009). Thus, ChAT-positive and NeuN-negative neurons were considered as gamma-motor neurons as we can see next to the white arrow in figures. Comparatively, in the AAV-NC + SCI and SCI group, neurons and motor neurons with edema and vacuolization were exhibited. Next, in the epicenter, few normal neurons and motor neurons in the anterior horn area were seen in rats after SCI. Only a few “neurons” with a quasi-circular shape and reduced size were found, which were considered to be abnormal cells caused by a microenvironmental disorder. Together, these results demonstrated the survival of motor neurons in the anterior horn of the spinal cord at 3 days after SCI. As seen in quantitative figure (Figure 2D), normal neurons in AAV-013017 + SCI group were most after SCI in rostral and caudal and epicenter segment significantly (*p* < 0.0001, *p* < 0.01, and *p* < 0.001). As seen in Supplementary Figures 3A,B. Representative image for neurons marked by NeuN and motor neurons marked by ChAt in the epicenter and caudal adjacent segment were shown.

Third, Nissl staining was performed at 3 days after SCI (Figure 2E). Similarly to IF, in the rostral and caudal adjacent segments, more Nissl bodies (indicated by red arrows) in the anterior horn area were seen in the AAV-013017 + SCI group than in control groups, showing higher cell activities after overexpression of target gene. Nissl bodies were normal in shape and blue-stained as blue chrysophoron. In the epicenter, only a few Nissl bodies could be found in the anterior horn in the AAV-013017 + SCI group. Instead, tissue edema, inflammation, poor cell activity, and severe cell damage were seen in the epicenters of injury in rats of all groups after SCI. As seen in quantitative figure (Figure 2F), These results were in line with IF staining, demonstrating different neuronal activities in all groups after SCI, which was likely related to the degree of injury, time of tissue collection, and peak time of neuronal apoptosis.

**FIGURE 3:**
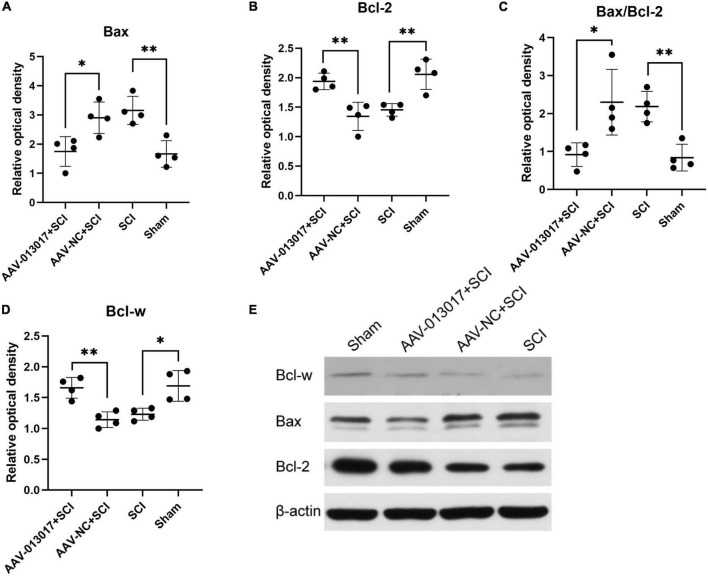
Expression of relative proteins in the spinal cord at 3 days after SCI. **(A)** The expression level of Bax was significantly altered in all groups at 3 days after SCI (*n* = 4 per group). **(B)** The expression level of Bcl-2 was significantly altered in all groups at 3 days after SCI (*n* = 4 per group). **(C)** The Bax/Bcl-2 ratio, which is an apoptosis-related indicator, was significantly altered in all groups at 3 days after SCI (*n* = 4 per group). **(D)** The expression level of Bcl-w was significantly altered in all groups at 3 days after SCI (*n* = 4 per group). **(E)** Protein expression levels of Bcl-w, Bcl-2, and Bax in spinal cord segments in all groups at 3 days after injury (*n* = 4 per group). Band optical densities were measured using Image J software. *Indicates *p* < 0.05; **indicates *p* < 0.01. Data are shown as means ± SD.

Lastly, WB was performed at 3 days after injury (Figures 3A–E). We found that the expression level of Bax—a pro-apoptotic protein—was the lowest in the AAV-013017 + SCI group compared with control groups (*p* < 0.05). The expression level of Bax was significantly increased in the SCI group compared with the sham group (*p* < 0.01). Additionally, we found that the expression level of Bcl-w—an anti-apoptotic protein—was the highest in the AAV-013017 + SCI group compared with control groups (*p* < 0.01). Compared with the AAV-NC + SCI group, the results showed that the expression level of Bcl-2, which is another anti-apoptotic protein, was markedly increased in the AAV-013017 + SCI group (*p* < 0.01). Compared with the SCI group, the results showed that the expression level of Bcl-2 was markedly increased in the sham group (*p* < 0.01). After calculating the Bax/Bcl-2 ratio—an apoptosis-related indicator—the results revealed that it was significantly reduced in the AAV-013017 + SCI group compared with the AAV-NC + SCI group (*p* < 0.05) and in the sham group compared with the SCI group (*p* < 0.01). These data demonstrated that the overexpression of the target gene downregulated the expression of pro-apoptotic proteins and upregulated the expression of anti-apoptotic proteins, suggesting an anti-apoptotic effect of rno-circRNA-013017.

### Inhibition of rno-circRNA-013017 on descending axonal degeneration

We used BDA, DTI, IF, and Nissl staining to identify the inhibiting effect of overexpressed rno-circRNA-013017 on descending axonal degeneration in the spinal anterior horn at 6 weeks after SCI.

**FIGURE 4:**
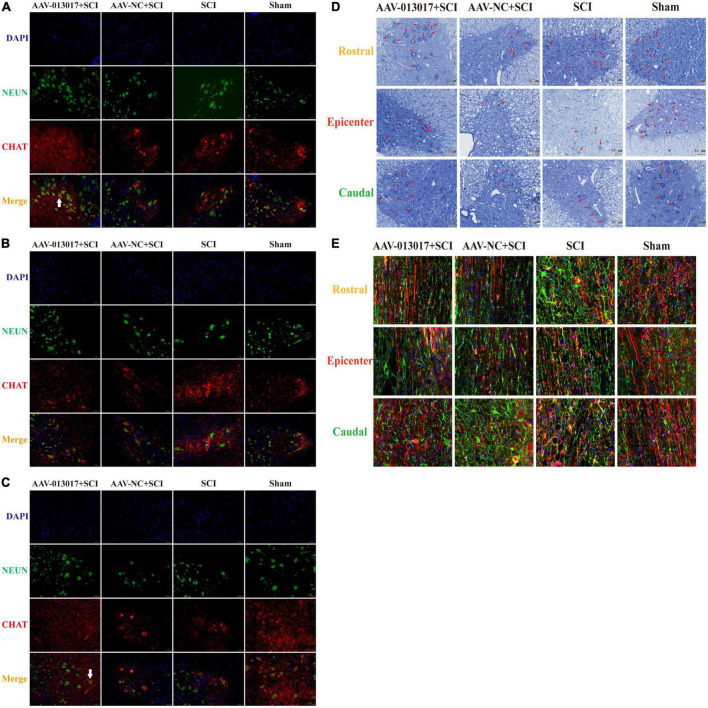
Inhibition by rno-circRNA-013017 of descending axonal degeneration at 6 weeks after SCI. **(A)** In the rostral adjacent segment, neurons marked by NeuN and motor neurons marked by ChAt in the anterior horn area were shown in all groups. The white arrow indicates a typical motor neuron with an irregular cell body, nucleus, axon, dendrite, and other cell structures (*n* = 5 per group). **(B)** In the epicenter, only a few normal neurons and motor neurons in the anterior horn area were seen in the three groups subjected to SCI (*n* = 5 per group). **(C)** In the caudal adjacent segment, neurons marked by NeuN and motor neurons marked by ChAt in the anterior horn area were observed in all groups. The white arrow indicates a typical motor neuron with an irregular cell body, nucleus, axon, dendrite, and other cell structures (*n* = 5 per group). **(D)** Nissl bodies indicated by red arrows in the anterior horn area were seen in all groups (*n* = 5 per group). **(E)** The density of descending axons of spared white matter marked by biotin dextran amine (BDA) (red lines) and astrocytes marked by GFAP (green points) are shown (*n* = 5).

First, IF was performed at 6 weeks after injury (Figures 4A–C). In the rostral and caudal adjacent segment, more neurons marked by NeuN and motor neurons marked by ChAt in the anterior horn area were seen in the AAV-013017 + SCI group than in the AAV-NC + SCI and SCI groups. The white arrow in the figure indicates a typical motor neuron with an irregular cell body, nucleus, axon, dendrite, and other cell structures. Next, in the epicenter, only a few normal neurons and motor neurons in the anterior horn area were seen in rats of all groups after SCI. Among these groups, relatively more neurons and motor neurons were found in the AAV-013017 + SCI group. Together, these results demonstrated the survival of motor neurons in the anterior horn of the spinal cord at 6 weeks after SCI.

Second, Nissl staining was conducted 6 weeks after injury (Figure 4D). Similarly to IF, more Nissl bodies (indicated by red arrows) in the anterior horn area were seen in the AAV-013017 + SCI group than control groups. Nissl bodies were normal in shape and blue-stained as blue chrysophoron. Comparatively, in the epicenter, only a few Nissl bodies could be found in the anterior horn in the AAV-013017 + SCI group. Among these groups, relatively more Nissl bodies were found in the AAV-013017 + SCI group. These results were in line with the IF staining, showing neuronal activity in all groups at 6 weeks after SCI, indicating that the overexpression of the target gene could protect more neuronal activity at 6 weeks after SCI. Besides, as seen in Supplementary Figure 4, in the epicenter of injury cord, the lesion area was relatively smallest and spared tissue was relatively largest in the AAV-013017 + SCI group at 6 weeks after SCI. As shown in Supplementary Figure 5, the number of motor neurons and Nissl bodies in IF and Nissl staining were calculated and depicted.

Third, BDA tracing was performed at 6 weeks after injury (Figure 4E). For comparisons among each segment of different groups, the density and number of descending axons marked by BDA (red lines) were the highest, while the density and number of astrocytes marked by glial fibrillary acidic protein (GFAP, green points) were the fewest in the sham group, which was in accordance with surgical operations and expectations of the experiment. Among the three groups subjected to SCI, the density and number of descending axons marked by BDA (red lines) were relatively higher, while the density and number of astrocytes marked by GFAP (green points) were relatively fewer in the AAV-013017 + SCI group, especially in the rostral and caudal adjacent segments. These results suggested the protective effects of the target gene on descending axons at 6 weeks after SCI.

**FIGURE 5:**
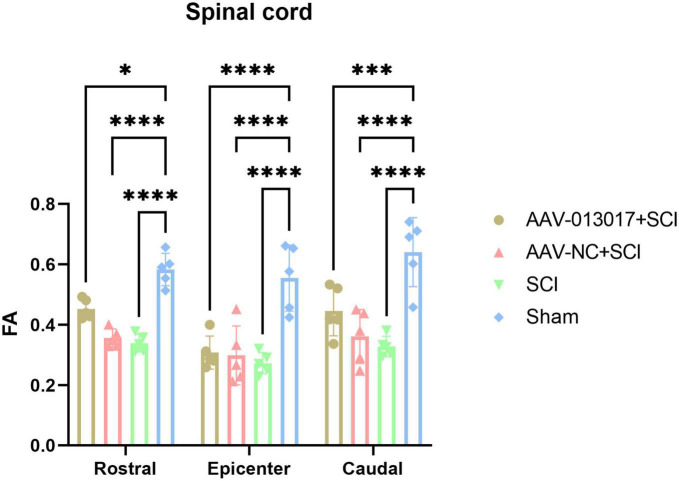
Diffusion tensor imaging (DTI) detection at 6 weeks after SCI. Fractional anisotropy (FA) was altered in different segments of different groups (*n* = 5 per group). *Indicates *p* < 0.05, ***indicates *p* < 0.001, ****indicates *p* < 0.0001.

**FIGURE 6:**
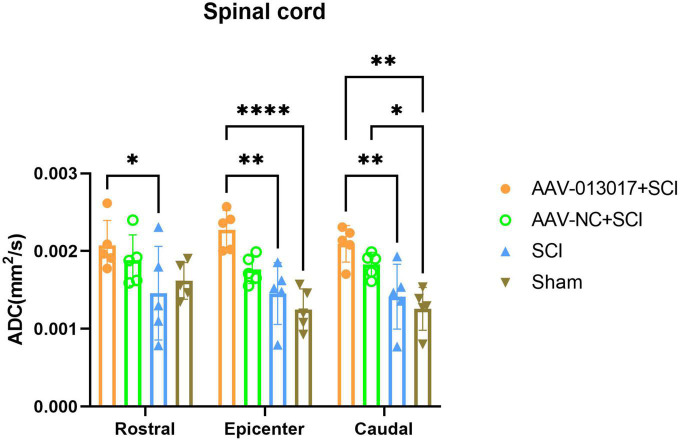
Diffusion tensor imaging detection at 6 weeks after SCI. Axial diffusivity (AD) was altered in different segments of different groups (*n* = 5 per group). Data are shown as means ± SD. *Indicates *p* < 0.05, **indicates *p* < 0.01, ****indicates *p* < 0.0001.

Fourth, a DTI scan was performed 6 weeks after injury (Figures 5, 6). For comparisons among each segment of different groups, except for the sham group, Fractional Anisotropy (FA) was higher in the AAV-013017 + SCI group than control groups, especially in the rostral and caudal adjacent segment, indicating the higher structural integrity of axons. However, no statistical significance was found for comparisons between the AAV-013017 + SCI group and controls. In terms of axial diffusivity (AD), AD was higher in the AAV-013017 + SCI group than control groups in all segments, indicating the higher diffusion properties of water molecules, which often stands for less tissue destruction and inflammation. However, no statistical significance was found for comparisons between the AAV-013017 + SCI group and controls. For comparisons among different segments of each group, FA was relatively higher in the rostral and caudal adjacent segments than in the epicenter, suggesting more damaged tissue in the epicenter, while no significant differences were found in terms of AD. Besides, seen in Supplementary Figure 6, detailed DTI images and image identification were shown which indicate the choice of ROI and the actual observation image cut position.

### Improvement by rno-circRNA-013017 of the locomotor function of rats after spinal cord injury

We used an open-field test (BBB score) and gait analysis to evaluate the locomotor function of rats in all groups after SCI.

**FIGURE 7:**
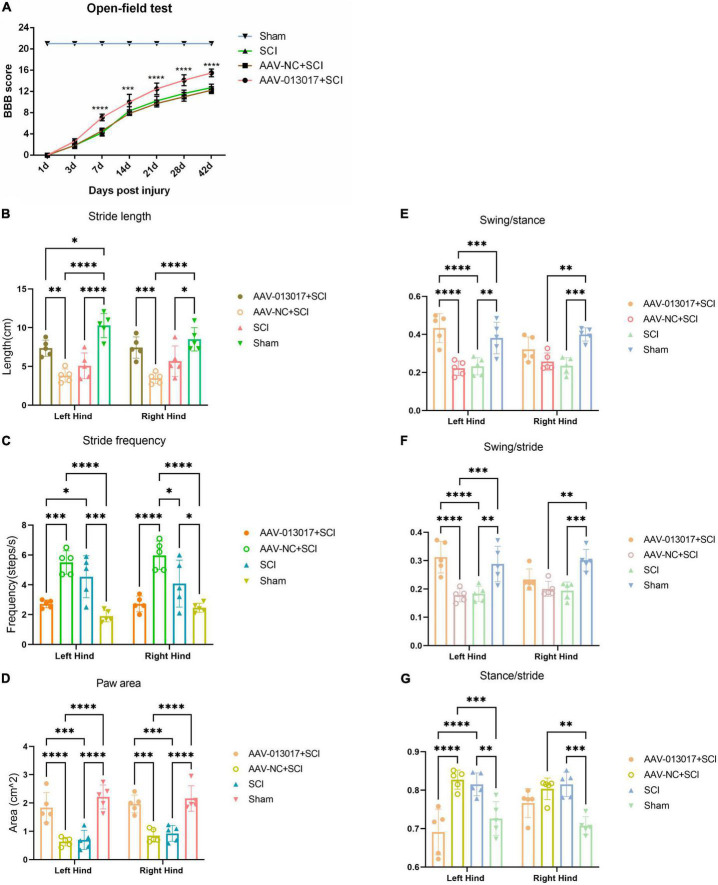
Improvement by rno-circRNA-013017 of the locomotor function of rats after SCI. **(A)** BBB scores among different groups were evaluated by an open field test (*n* = 5 per group). ***Indicates *p* < 0.001 for the AAV-013017 + SCI group vs. AAV-NC + SCI group; ****indicates *p* < 0.0001 for the AAV-013017 + SCI group vs. AAV-NC + SCI group; **(B–G)** Gait analysis using an automated treadmill with relative indexes: Stride length, stride frequency, paw area, swing/stance, swing/stride, and stance/stride. *Indicates *p* < 0.05, **indicates *p* < 0.01, and ***indicates *p* < 0.001, ****indicates *p* < 0.0001. Data are shown as means ± SD.

First, on the day of SCI, all rats lost the locomotor function of their lower limbs. We then found that the BBB scores of rats in the AAV-013017 + SCI group were significantly higher than in the two control groups after SCI, especially at 7, 14, 21, 28, and 42 days after SCI, suggesting better functional recovery after overexpression of the target gene (Figure 7A).

Second, gait analysis was performed at 6 weeks after injury (Figures 7B–G). The results showed that the stride length and paw area were greater, while the stride frequency was lower, for both of the hind limbs of rats in the AAV-013017 + SCI group than control groups, with statistical significance. Moreover, the swing/stance was significantly higher, while the swing/stride and stance/stride were significantly lower, especially for the left hind limbs, of rats in the AAV-013017 + SCI group than in the control groups. Besides, seen in Supplementary Figure 7, the gait summary and footprint pictures of rats for different groups were shown, which suggested that distances between forelimb and hindlimb of rats were smallest in AAV-013017 + SCI and sham group. Together, these results showed that rats in the AAV-013017 + SCI group were similar to the rats in sham groups, indicating a locomotor improvement of rats after gene therapy.

## Discussion

In summary, based on our previous work, we found that five specific circRNAs were significantly downregulated after SCI. In this study, we initially used bioinformatics methods, literature retrieval, and the luciferase reporter gene technique to preliminarily speculate that rno-circRNA-013017, our target circRNA, is likely to inhibit apoptosis *via* negatively regulating its target gene—rno-miR-16-5p. After verifications *via* various relative experiments, taken together, the results finally showed that the overexpression of rno-circRNA-013017 could inhibit motor neuron apoptosis in the anterior horn area and reduce descending axonal degeneration, thus leading to the locomotor recovery of rats after SCI. In reviewing the literature, the present study offers the first comprehensive functional verifications of specific circRNA in rat models after traumatic SCI, providing strong evidence for further investigations of and translational research on SCI.

Most of the human genome is composed of ncRNAs (Cimmino et al., 2005; Yang et al., 2017; Yin et al., 2020), which are widely involved in physiological and pathological activities and are closely associate with various diseases (Anastasiadou et al., 2018; Dimartino et al., 2018), such as cancer, cardiovascular diseases, and neurological disorders. Compared to miRNAs and lincRNAs, circRNAs are more stable because of their unique covalently closed loop and specific tertiary structures, which offer more possibilities to act as ideal biomarkers or novel therapeutic targets. According to a lot of studies, circRNA is involved in gene regulation at the transcriptional or post-transcriptional level. Generally, there are three main functions of circRNAs:1) miRNA sponge: namely some circRNAs have lots of miRNA response elements (MREs), which can form RNA-induced silencing complexes with AGO proteins, bind as competing endogenous RNAs (ceRNAs), inhibit the function of target miRNAs, and regulate downstream mRNA expression; 2) Transcriptional regulation: Some intron or exon-intron circRNAs are involved in gene expression as post-transcriptional regulatory factors and can regulate the transcription and translation process of linear RNA, however, the mechanism remains to be further clarified; 3) Endogenous translation: circRNAs, as a non-coding RNA, has long been thought to have no coding capability. but recent studies have shown that it is possible for circRNA to code peptides. In terms of SCI, several studies (Qin et al., 2019; Wu et al., 2019; Liu Y. et al., 2020; Peng et al., 2020) have found using a microarray or sequencing that the expression profiles of circRNAs were significantly altered in rat models at different time points after SCI and constructed a circRNA–miRNA–mRNA network with bioinformatics tools. In these studies, functional verifications of candidate circRNA were merely preliminarily predicted, rather than validated by functional experiments. Recently, one study (Zhao J. et al., 2020) involving functional explorations of circRNA showed that circ-HIPK3 relieved neuronal cell apoptosis by regulating the miR-588/DPYSL5 axis in SCI. However, this study found the role of a novel circ-HIPK3/miR-558/DPYSL5 axis in SCI mainly *via* experimental verifications in PC12 cell lines with the treatment of CoCl2, lacking validations of overall functions *in vivo*. In the present study, we performed functional experiments of rno-circRNA-013017, which promoted the locomotor recovery of rats subjected to SCI.

In this investigation, we found that the overexpression of rno-circRNA-013017 could inhibit the apoptosis of motor neurons in the spinal anterior horn, especially in the rostral and caudal adjacent segments at 3 days after SCI. Nevertheless, in the epicenter, there were no significant differences of neuron apoptosis among the three groups subjected to SCI, which was likely related to the peak time of neuronal apoptosis and degree of injury. Referring to a system review (Hassannejad et al., 2018), TUNEL staining of the sections from the ventral horn revealed the presence of apoptotic neurons at 4 h post injury. After that, the number of apoptotic neurons was maximized 8 h post injury and subsequently decreased in the lesion area—namely, the first peak of apoptosis (Liu et al., 1997). At 3 days post injury, no discernable apoptotic activity was shown in the immediate zone of the SCI. However, the maximum apoptosis in neurons was detected in approximately 6–7 mm rostral and caudal to the epicenter—namely, the second peak of apoptosis (Yong et al., 1998). As a result, our findings of TUNEL staining at 3 days after SCI were in line with the second peak of neuron apoptosis in rats. As for the degree of injury, T10 lesion areas were directly subjected to moderate contusion (Khuyagbaatar et al., 2015). Therefore, many motor neurons and axons were immediately damaged without apoptosis by mechanical injury in the epicenter, while more motor neurons survived in T9 and T11 at 3 days after SCI. This evidence explains why, for different segments of each group, more neuronal apoptosis (TUNEL) and motor neuronal survival (IF and Nissl) were presented in T9 and T11 than T10 after SCI.

For clinical translation, first, apoptosis of neuronal cells after spinal cord injury appears in early stages. Thus, interventions must be at or before this stage. Besides, we administered the injection immediately after spinal cord injury in rats in pre-experiments. We found unsatisfactory transfection results because edema and hemorrhage of spinal cord tissue and high tension can prevent AAV transfection and even lead to injection failure. Thus, to verify the biological function of this gene, we injected before the damage. When we performed the damage, the cell transfection efficiency peaked. Our next step will be to use post-injury intraperitoneal or intravenous AAV for clinical translational studies. Pre-protection is not suitable for standard clinical intervention.

## Conclusion

In conclusion, rno-circRNA-013017 was significantly downregulated in rats after injury. After overexpression, this target gene exhibited an inhibiting effect on motor neuron apoptosis in the anterior horn and descending axonal degeneration, protecting and preserving more motor neurons and axons, and finally promoting the locomotor function of rats after SCI.

However, several questions still need to be answered. First, our future work will include rescue experiments on rno-circRNA-013017 and its target genes to verify their relationship *in vitro* and *in vivo*. Namely, we will verify the rno_circRNA_013017/rno-miR-16-5p/bcl-2 pathway in cell lines and then perform the animal experiments. Furthermore, more work on the drug dose, administration method, and side effects of rno-circRNA-013 017 should be conducted before applying it to clinical patients. Next, we strongly recommend that future research should focus on different animal models and types of SCI to explore the functions of specific circRNAs. Last but not least, TUNEL positive cells and its data presented supports the conclusion but it fails to identity the cell-types in the spinal cord. There are more non-neuronal cells in the spinal cord that neuronal population. It will be very important to know the exact cell type(s) being targeted by rno-circRNA-013017 by showing colocalizations with cell-specific antibodies. We will make this attempt in the future.

## Data availability statement

The original contributions presented in this study are included in the article/Supplementary material, further inquiries can be directed to the corresponding authors.

## Ethics statement

This animal study was reviewed and approved by the Institutional Animal Care and Use Committee of Capital Medical University, Beijing, China.

## Author contributions

CQ, YL, and P-PX performed the research. J-JL and FG designed the research study. XZ and ZT contributed to the essential reagents or tools. J-YL, Y-LJ, and FB analyzed the data. CQ wrote the manuscript. L-XZ and YY revised the manuscript. All authors read and approved the final manuscript.
