# Supplementary material for: Inhibition by rno-circRNA-013017 of the apoptosis of motor neurons in anterior horn and descending axonal degeneration in rats after traumatic spinal cord injury
Source: Front Neurosci. 2022 Dec 15;16:1065897. doi: 10.3389/fnins.2022.1065897 (PMC9797719; doi:10.3389/fnins.2022.1065897)
Supplement: Supplementary file 1 [file Data_Sheet_1.pdf]

Supplementary Material

Supplementary Figure 1. The gene array analysis information map of bioinformatics screening and prediction for selecting circular RNA (circRNA). (A) The scatter plot showed the differences in circRNA expression between the SCI and sham groups. The values of the X and Y axes in the scatter plot are the normalized signal values of the samples (log2 scaled) or the averaged normalized signal values of groups of samples (log2 scaled). The green lines are fold change lines. The circRNAs above the top green line and below the bottom green line indicated more than 2-fold changes of circRNAs between the two compared samples. (B) Volcano plots show the differentially expressed circRNAs with statistical significance (fold change⩾2; $P<0.05$) The vertical lines correspond to 2.0-fold up and down, respectively, and the horizontal line represents a P of 0.05; the red point in the plot represents differentially expressed circRNAs with statistical significance. (C) The circRNA/miRNA/mRNA network analysis includes the 5 circRNAs, 60 miRNAs and 253 mRNAs (Nodes with red color are miRNAs; nodes with light-blue color are mRNAs; nodes with brown color are circRNAs).

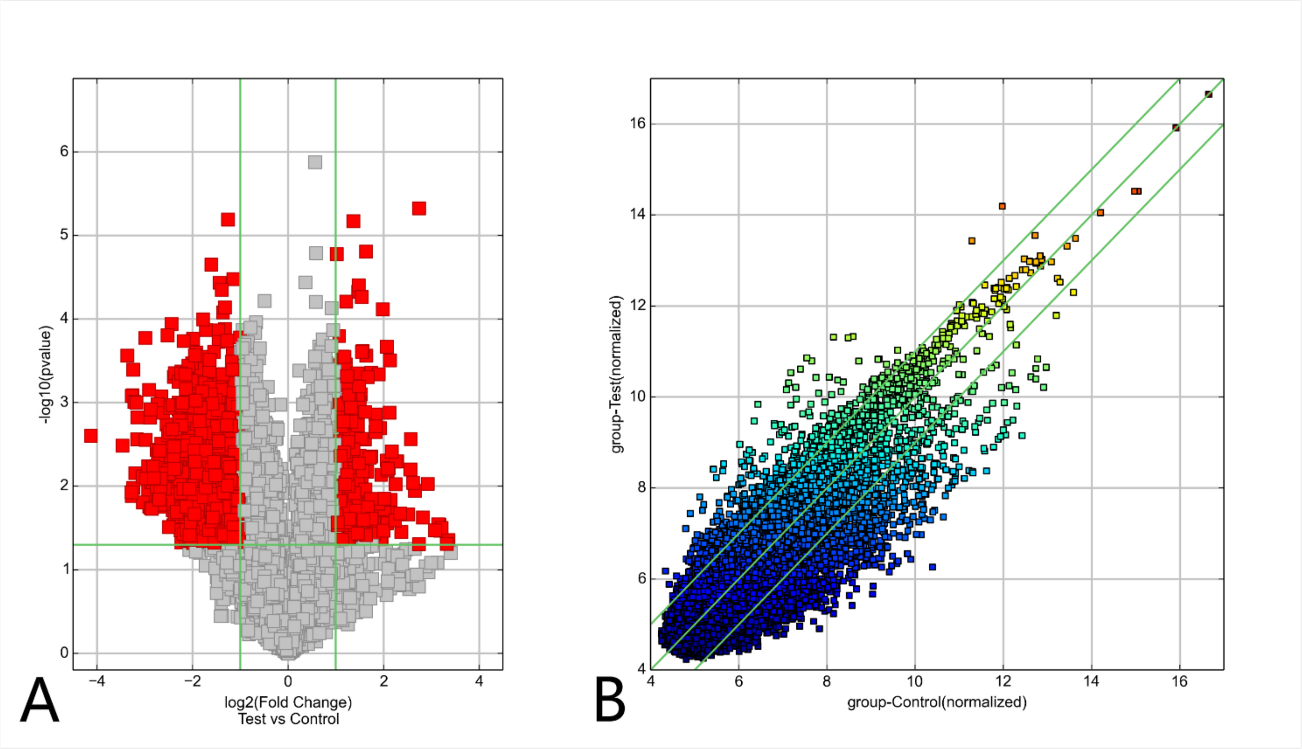
A
-log10(pvalue)
0
1
2
3
4
5
6
−4
−2
0
2
4
log2(Fold Change)
Test vs Control
B
group-Test(normalized)
4
6
8
10
12
14
16
group-Control(normalized)

C

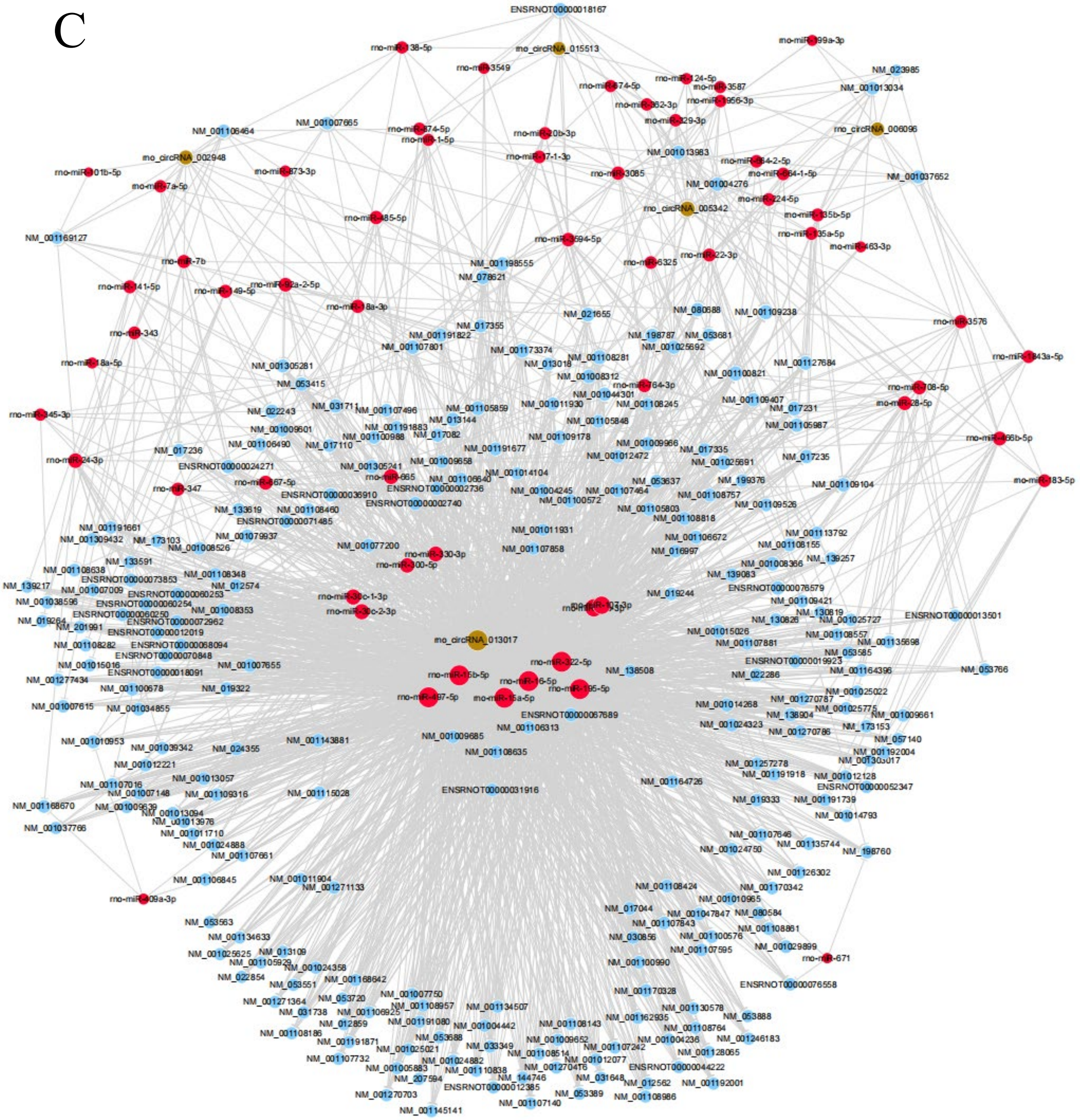

Supplementary Figure 2. Selection of target circular RNA (circRNA) and study design illustration. (A) Bioinformatics showed that rno_circRNA_013017 and its target gene rno-miR-16-5p matched to a high degree. (B) A schematic of the base-pair regions of the rno-miR-16-5p 3'UTR and the predicted binding site for the target gene—rno_circRNA_013017—is shown, with the wild-

type and mutant seed sequences listed below. (C) The repressive effect of rno_circRNA_013017 on the activity of the site of rno-miR-16-5p 3′UTR, measured by a luciferase reporter assay, indicating that rno_circRNA_013017 directly targeted rno-miR-16-5p. *** indicates p < 0.001. (D) Study design illustration for apoptosis detection. (E) Study design illustration for the detection of axonal degeneration.

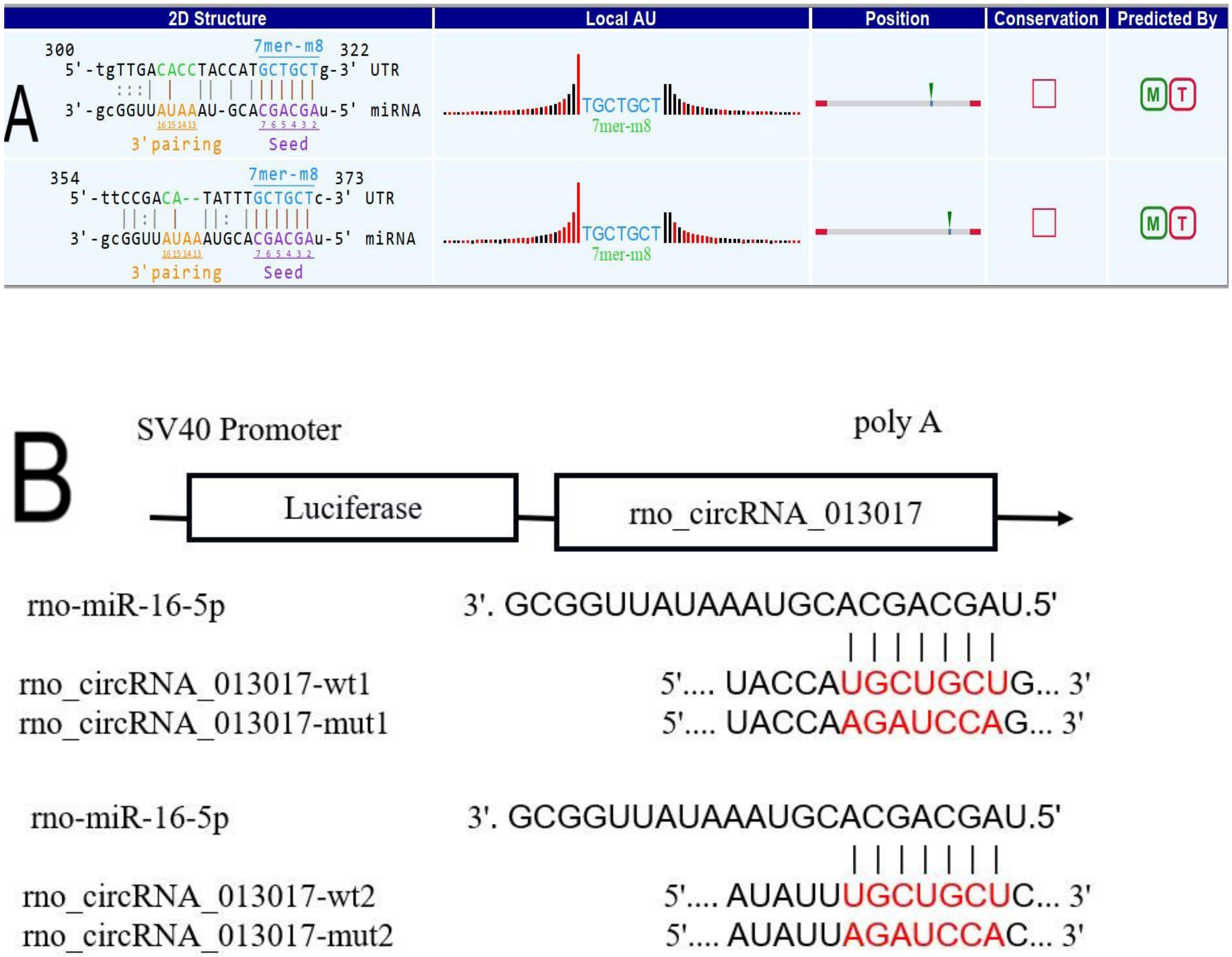

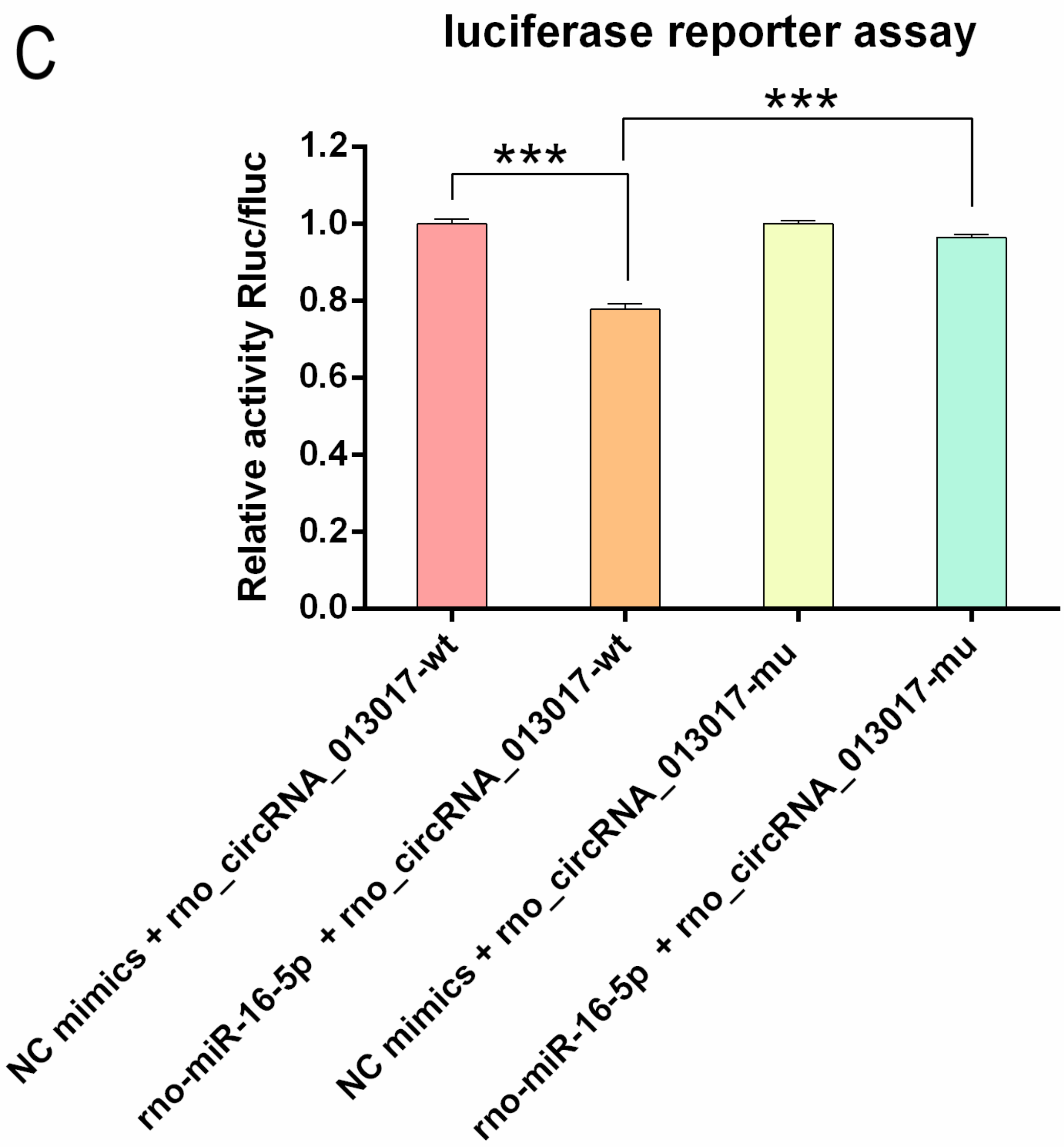
C
luciferase reporter assay
***
***
Relative activity Rluc/fluc
1.2
1.0
0.8
0.6
0.4
0.2
0.0
NC mimics + rno_circRNA_013017-wt
rno-miR-16-5p + rno_circRNA_013017-wt
NC mimics + rno_circRNA_013017-mu
rno-miR-16-5p + rno_circRNA_013017-mu

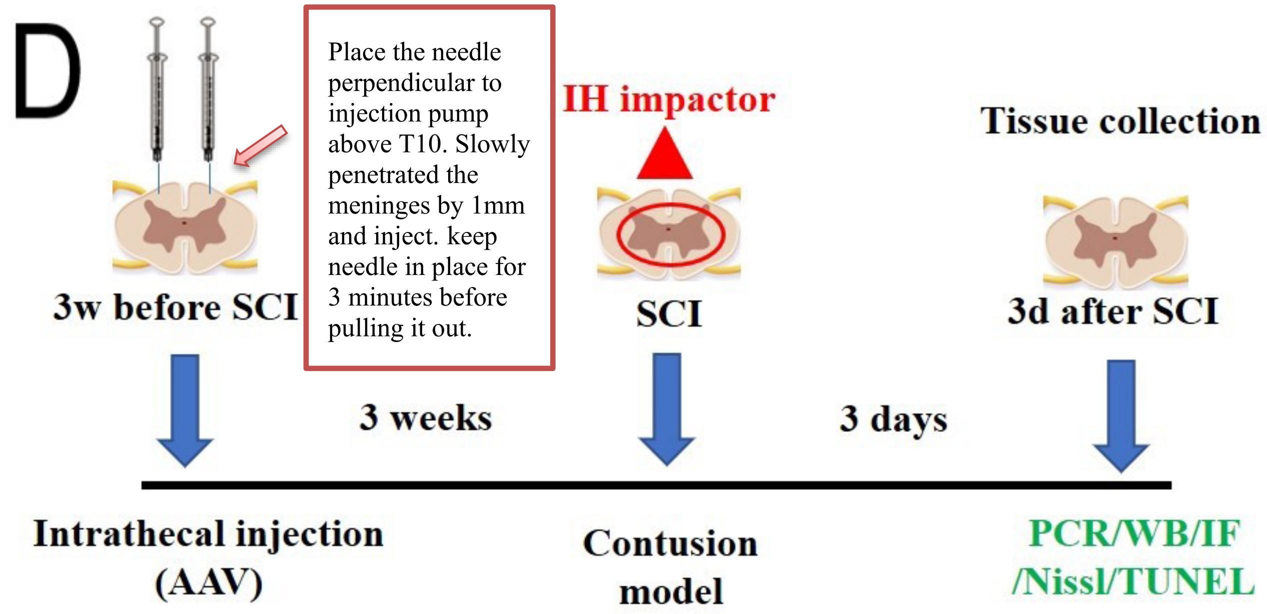
D
Place the needle perpendicular to injection pump above T10. Slowly penetrated the meninges by 1mm and inject. keep needle in place for 3 minutes before pulling it out.
IH impactor
Tissue collection
3w before SCI
SCI
3d after SCI
3 weeks
3 days
Intrathecal injection (AAV)
Contusion model
PCR/WB/IF /Nissl/TUNEL

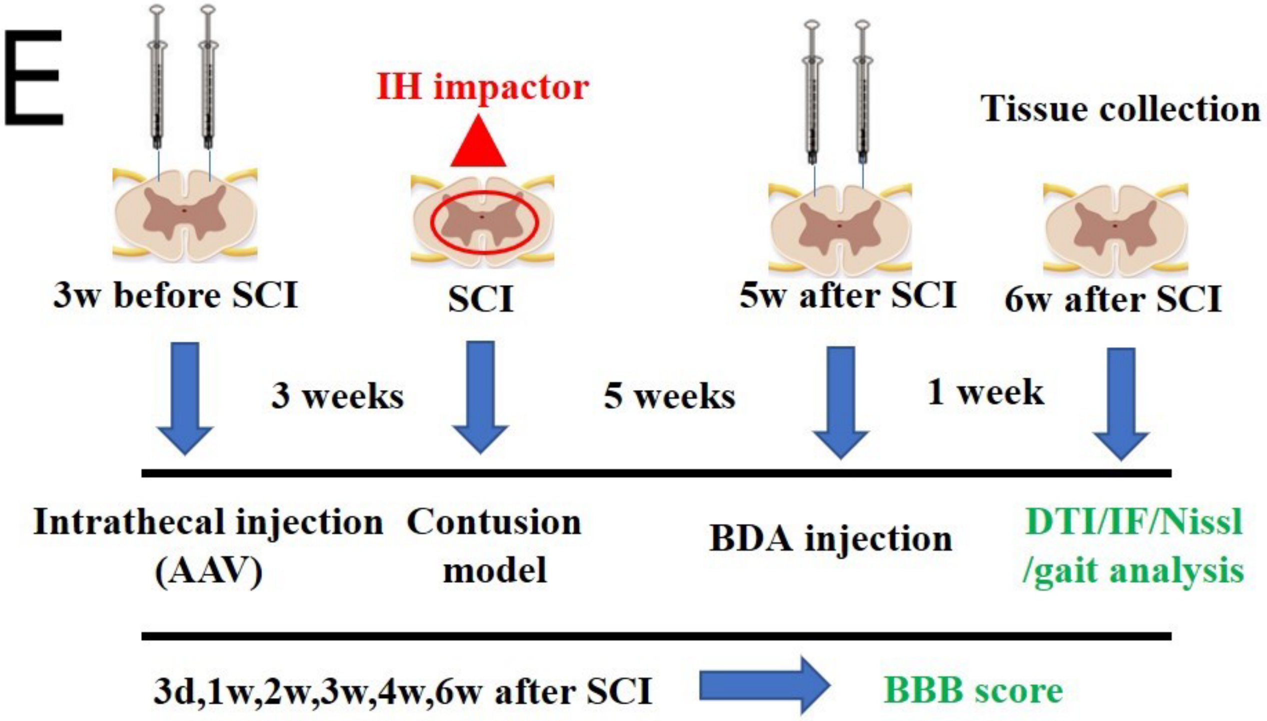
E
IH impactor
Tissue collection
3w before SCI
SCI
5w after SCI
6w after SCI
3 weeks
5 weeks
1 week
Intrathecal injection (AAV)
Contusion model
BDA injection
DTI/IF/Nissl /gait analysis
3d,1w,2w,3w,4w,6w after SCI
BBB score

Supplementary Table 1. Relative expression of target gene in 293T cell line.

Sample Name	GAPDH Average CT	rno_circRNA_013017 Average CT	ΔCT	ΔΔCT	$2^{-\Delta\Delta CT}$
293T-control	17.93±0.3	28.78±0.23	10.85±0.38	0±0.38	1(0.77～1.3)
293T-pK25ssAAV	18.02±0.16	28.94±0.19	10.91±0.24	0.06±0.24	0.96(0.81～1.14)
293T-C13017	18.23±0.27	19.03±0.07	0.8±0.28	-10.06±0.28	1065.42(877.5～1293.59)

Supplementary Figure 3. Representative images for Fig. 2D. (A) Representative image for neurons marked by NeuN and motor neurons marked by ChAt in the epicenter. (B) Representative image for neurons marked by NeuN and motor neurons marked by ChAt in the caudal adjacent segment.

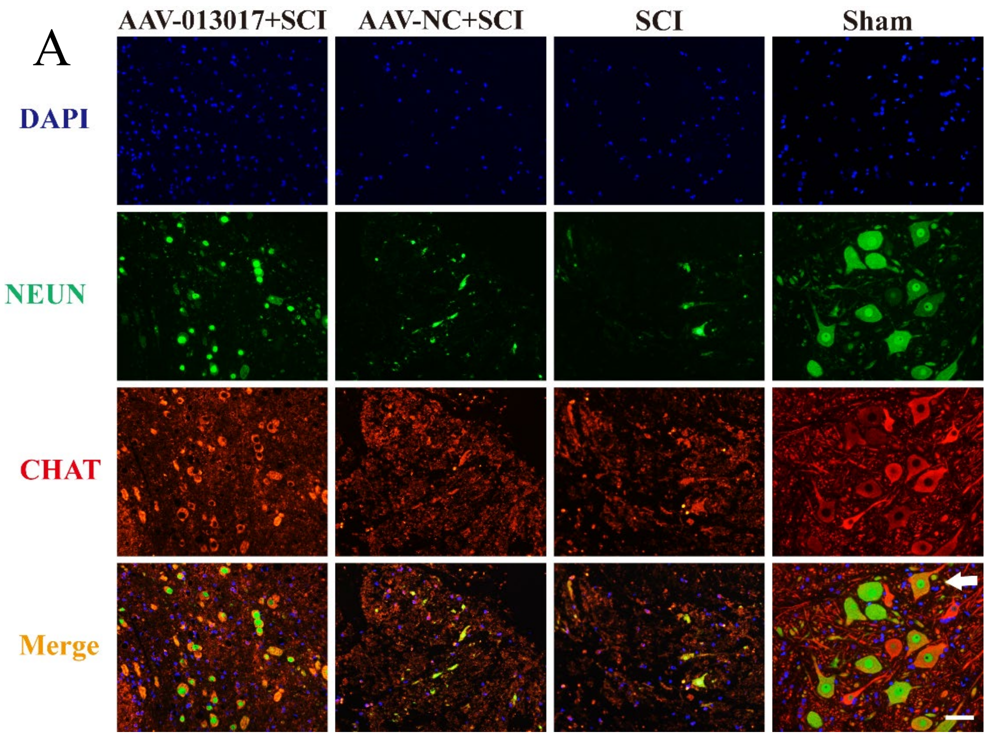

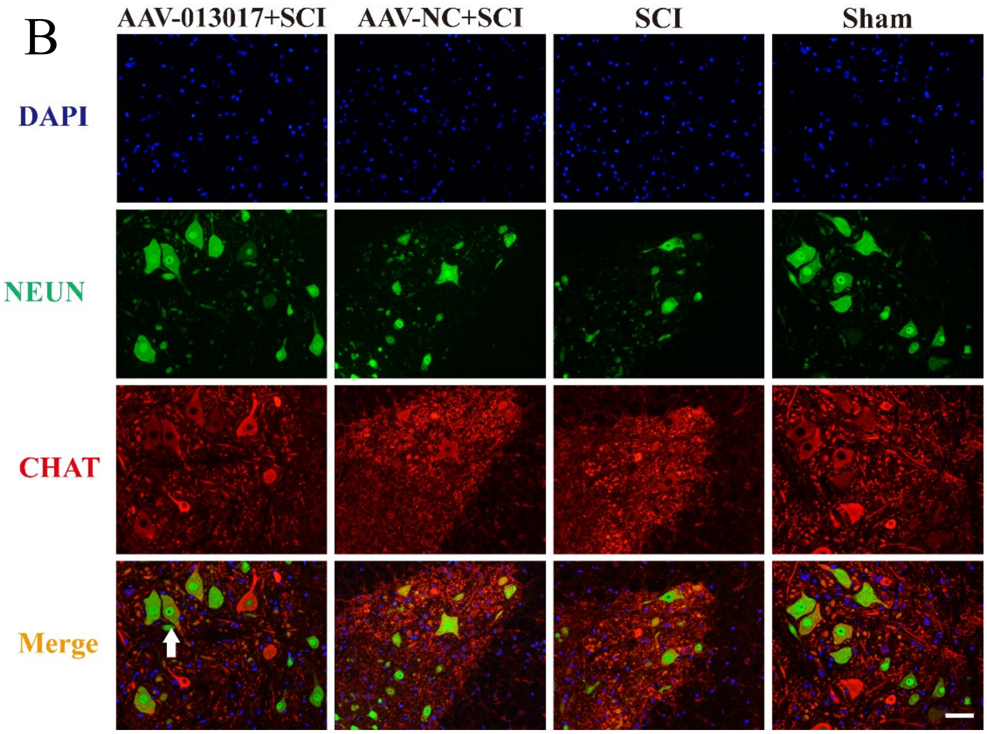

Supplementary Figure 4. **Representative images for Fig. 4D.** (A) AAV-013017+SCI group. (B) AAV-NC+SCI group. (C) SCI group. (D) Sham group.

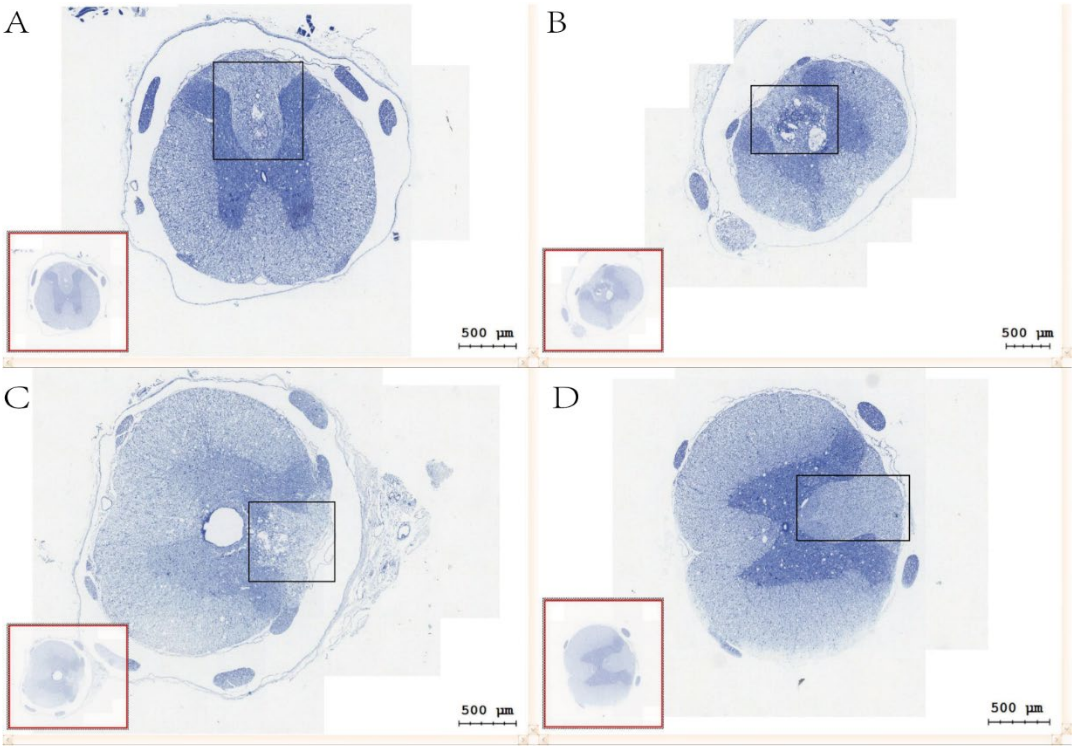

Supplementary Figure 5. Quantitative figure for figure4A-D. (A) Quantitative figure for motor neurons marked by NeuN/ChAT in IF in figure 4A-C. (B) Quantitative figure for Nissl's bodies marked by Nissl's staining in figure 4D. **** indicates p < 0.0001. ** indicates p < 0.01.

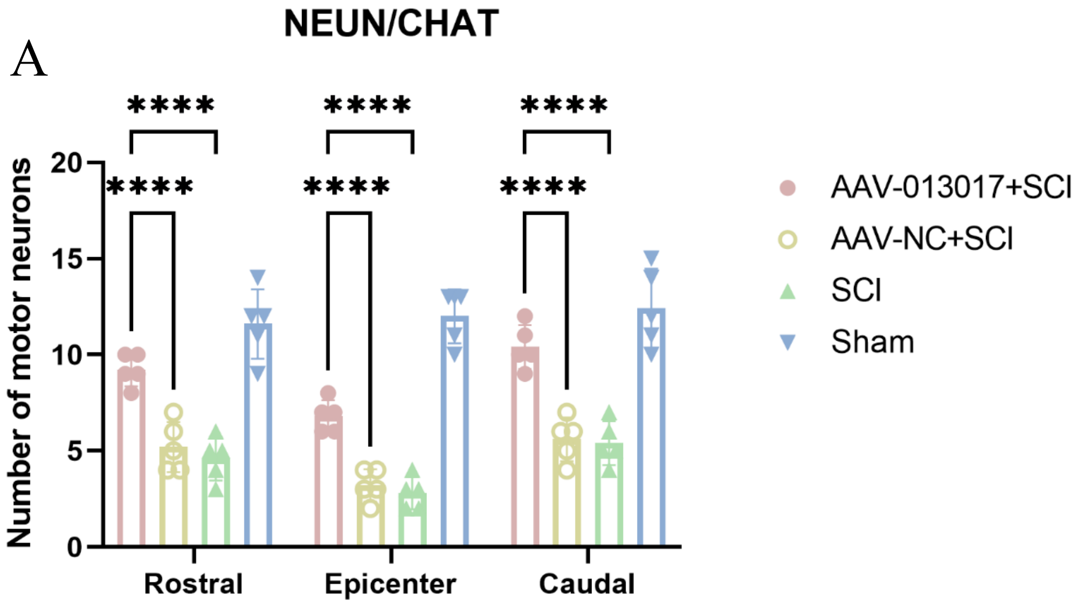

B

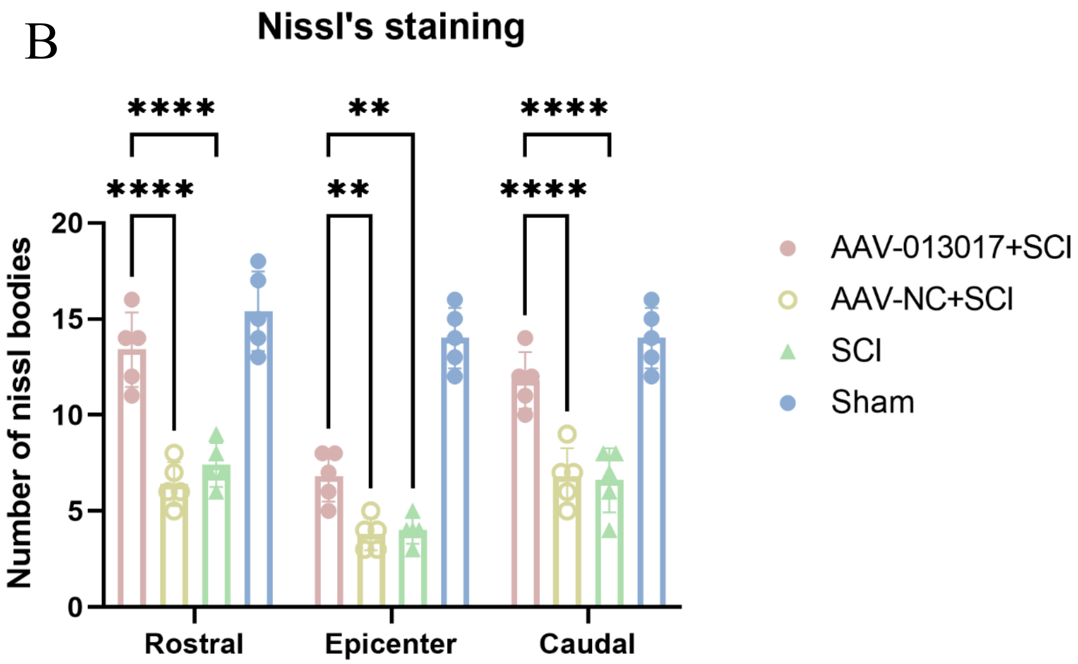

Supplementary Figure 6. Quantitative figure for Fig. 5-6. Detailed DTI images and image identification, and indicate the choice of ROI and the actual observation image cut position. (A) Detailed DTI images of spinal cord and image identification for AAV-013017+SCI group. (B-C) The actual observation image cut position of FA and ADC for AAV-013017+SCI group. (D) Detailed DTI images of spinal cord and image identification for AAV-NC+SCI group. (E-F) The actual observation image cut position of FA and ADC for AAV-NC+SCI group. (G) Detailed DTI images of spinal cord and image identification for SCI group. (H-I) The actual observation image cut position of FA and ADC for SCI group. (J) Detailed DTI images of spinal cord and image identification for Shame group. (K-L) The actual observation image cut position of FA and ADC for Shame group.

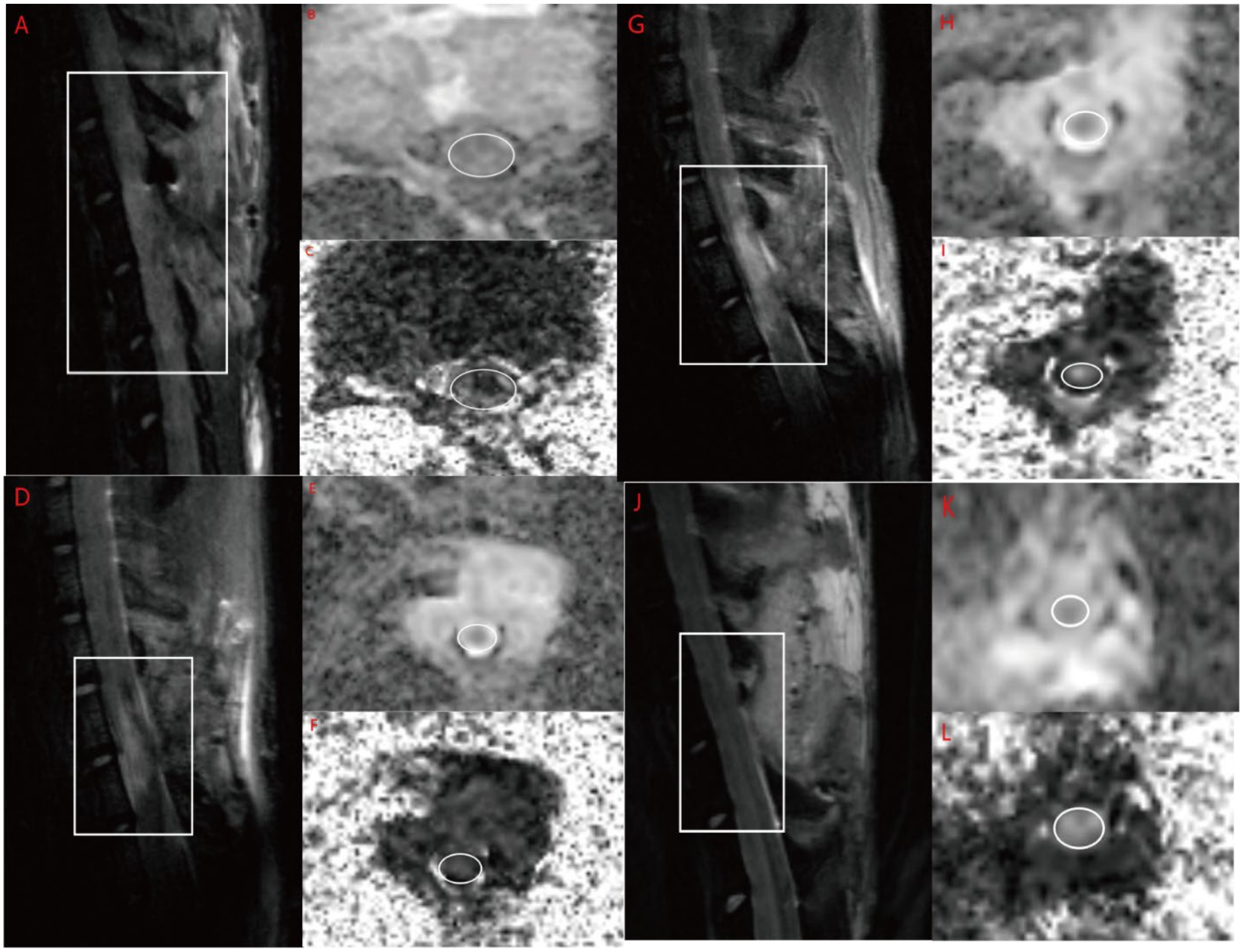

Supplementary Figure 7. Gait summary and footprint pictures of rats for figure7B-G. (A-B) AAV-013017+SCI group. (C-D) AAV-NC+SCI group. (E-F) SCI group. (G-H) Sham group.

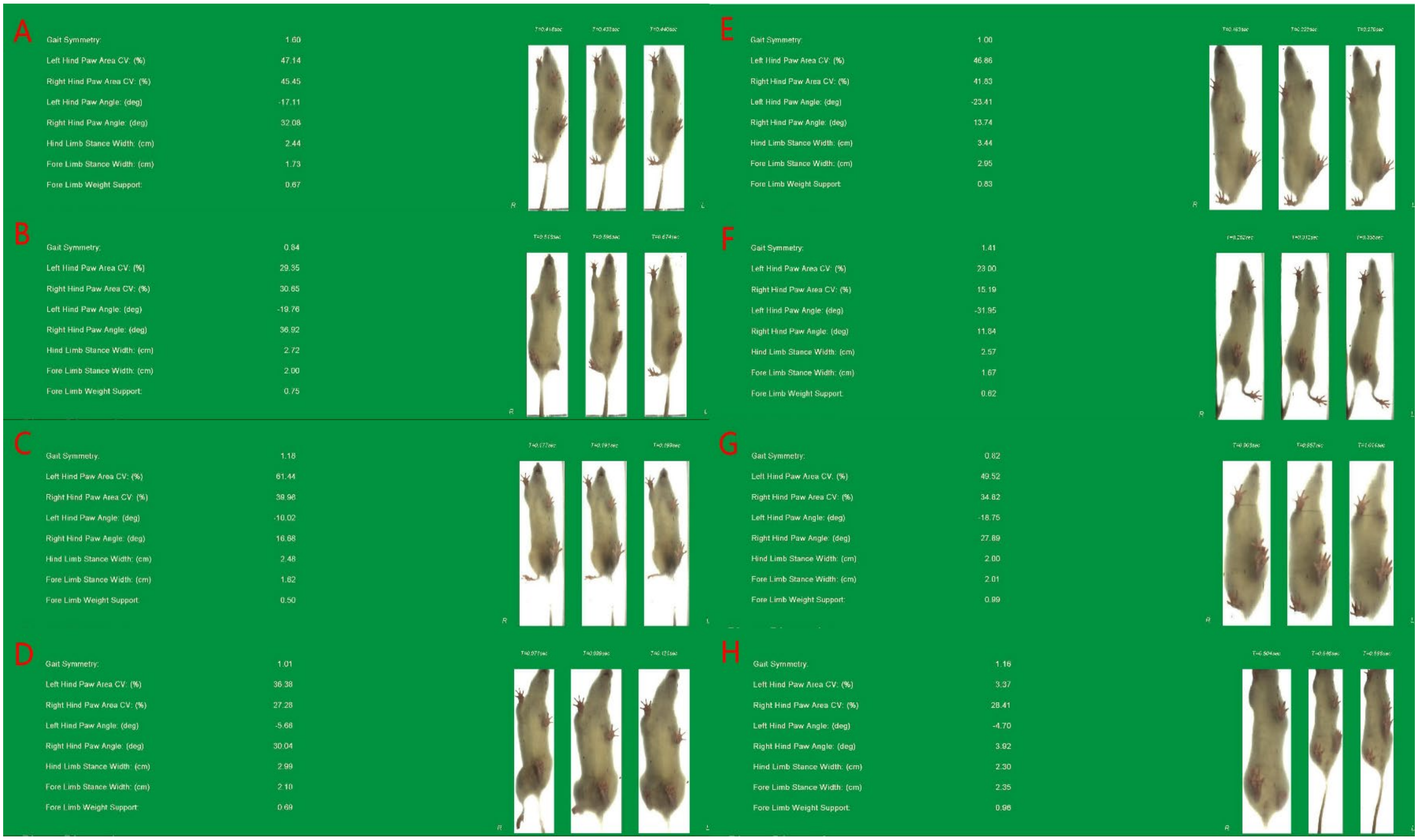
A
Gait Symmetry: 1.60
Left Hind Paw Area CV: (%) 47.14
Right Hind Paw Area CV: (%) 45.45
Left Hind Paw Angle: (deg) -17.11
Right Hind Paw Angle: (deg) 32.08
Hind Limb Stance Width: (cm) 2.44
Fore Limb Stance Width: (cm) 1.73
Fore Limb Weight Support: 0.67
B
Gait Symmetry: 0.84
Left Hind Paw Area CV: (%) 29.35
Right Hind Paw Area CV: (%) 30.65
Left Hind Paw Angle: (deg) -19.76
Right Hind Paw Angle: (deg) 36.92
Hind Limb Stance Width: (cm) 2.72
Fore Limb Stance Width: (cm) 2.00
Fore Limb Weight Support: 0.75
C
Gait Symmetry: 1.18
Left Hind Paw Area CV: (%) 61.44
Right Hind Paw Area CV: (%) 39.98
Left Hind Paw Angle: (deg) -10.02
Right Hind Paw Angle: (deg) 16.68
Hind Limb Stance Width: (cm) 2.48
Fore Limb Stance Width: (cm) 1.62
Fore Limb Weight Support: 0.50
D
Gait Symmetry: 1.01
Left Hind Paw Area CV: (%) 36.38
Right Hind Paw Area CV: (%) 27.28
Left Hind Paw Angle: (deg) -5.68
Right Hind Paw Angle: (deg) 30.04
Hind Limb Stance Width: (cm) 2.99
Fore Limb Stance Width: (cm) 2.10
Fore Limb Weight Support: 0.69
E
Gait Symmetry: 1.00
Left Hind Paw Area CV: (%) 46.86
Right Hind Paw Area CV: (%) 41.83
Left Hind Paw Angle: (deg) -23.41
Right Hind Paw Angle: (deg) 13.74
Hind Limb Stance Width: (cm) 3.44
Fore Limb Stance Width: (cm) 2.95
Fore Limb Weight Support: 0.83
F
Gait Symmetry: 1.41
Left Hind Paw Area CV: (%) 23.00
Right Hind Paw Area CV: (%) 15.19
Left Hind Paw Angle: (deg) -31.95
Right Hind Paw Angle: (deg) 11.84
Hind Limb Stance Width: (cm) 2.57
Fore Limb Stance Width: (cm) 1.67
Fore Limb Weight Support: 0.62
G
Gait Symmetry: 0.82
Left Hind Paw Area CV: (%) 49.52
Right Hind Paw Area CV: (%) 34.82
Left Hind Paw Angle: (deg) -18.75
Right Hind Paw Angle: (deg) 27.89
Hind Limb Stance Width: (cm) 2.00
Fore Limb Stance Width: (cm) 2.01
Fore Limb Weight Support: 0.99
H
Gait Symmetry: 1.16
Left Hind Paw Area CV: (%) 3.37
Right Hind Paw Area CV: (%) 28.41
Left Hind Paw Angle: (deg) -4.70
Right Hind Paw Angle: (deg) 3.92
Hind Limb Stance Width: (cm) 2.30
Fore Limb Stance Width: (cm) 2.35
Fore Limb Weight Support: 0.96
